# JMbdirect: joint modeling with semi-parametric link functions for bidirectional feedback in longitudinal-survival data

**DOI:** 10.1186/s12874-026-02955-5

**Published:** 2026-08-01

**Authors:** Atanu Bhattacharjee

**Affiliations:** Population Health and Genomics, School of Medicine, University of Dundee, Dundee, UK

**Keywords:** Joint modeling, Bidirectional feedback, Semi-parametric links, Dynamic prediction, Electronic health records

## Abstract

**Background:**

Joint models of longitudinal and time-to-event data are essential for dynamic prediction in chronic disease research, but standard formulations typically assume linear biomarker–hazard associations and unidirectional influence from biomarkers to events. These assumptions are often violated in multimorbidity and oncology, where clinical events alter subsequent biomarker trajectories and where biomarker–risk relationships are non-linear or threshold-like. Methods that relax both assumptions simultaneously, and that are accessible to applied researchers, are currently lacking.

**Methods:**

We extend the joint modeling framework through the JMbdirect package by introducing semi-parametric association structures based on penalized splines and neural additive components, and by embedding explicit bidirectional feedback between events and biomarkers via post-event level shifts and slope changes in the longitudinal trajectory. Estimation is supported via penalized maximum likelihood with Laplace approximation and a Bayesian alternative using Hamiltonian Monte Carlo. Performance was assessed in simulation studies crossing four design factors (form of the association function, presence and magnitude of feedback, single versus competing events, and baseline hazard specification) at sample sizes $n \in \{200, 500, 1000\}$, and in an applied analysis of the primary biliary cirrhosis (PBC) cohort.

**Results:**

Incorporating feedback and flexible links improved discrimination and calibration relative to classical linear specifications, particularly under non-linear risk mechanisms. In the representative competing-risks scenario (*n=500*), the spline-with-feedback estimator attained AUC@5 of 0.85 and an integrated Brier score of 0.134 with near-nominal 95% coverage, compared with AUC@5 of 0.72, an integrated Brier score of 0.168 and 88% coverage for the linear model without feedback; calibration slopes were 0.99 and 0.74, respectively. Feedback parameters were recovered with negligible bias at moderate and strong magnitudes. In the PBC application, the spline-based model identified a threshold effect at approximately 3 mg/dL of serum bilirubin and improved cross-validated discrimination (AUC@5 0.78 versus 0.71 for the linear specification).

**Conclusions:**

Flexible association structures and explicit bidirectional feedback yield more accurate and more interpretable dynamic predictions than classical joint models, with manageable computational cost. By combining these methodological advances with an open-source R package and a publicly available dashboard interface, this work supports the translation of joint modeling into personalized risk prediction and clinical decision support in multimorbid populations.

**Supplementary Information:**

The online version contains supplementary material available at https://doi.org/10.1186/s12874-026-02955-5.

## Background

Chronic diseases frequently exhibit *bidirectional relations*, where one condition influences and is influenced by another over time. For example, type 2 diabetes substantially increases the risk of cardiovascular disease (CVD), while the onset of CVD can worsen glycaemic control and accelerate diabetes complications, creating a feedback loop that compounds risk [1]. Similarly, chronic kidney disease (CKD) may arise as a complication of diabetes but also independently predicts cardiovascular mortality, reinforcing the cyclical interplay between these conditions [2].

These interdependencies are central to the study of *multimorbidity*, defined as the coexistence of two or more chronic conditions within an individual [3]. Traditional epidemiological approaches that treat diseases in isolation risk underestimating the true burden of multimorbidity and misinforming treatment strategies [4]. From a methodological perspective, recognising bidirectional relations is highly relevant for *time-to-event analyses*. Joint models of longitudinal and survival data, as well as multi-state frameworks, provide statistical tools to capture these complex dynamics by simultaneously modelling disease trajectories and event risks [5, 6]. Such approaches enable *dynamic prediction* of future occurrences (e.g., hospitalisation, progression, or death), accounting for how one disease modifies the risk profile of another [7]. In multimorbidity research, this perspective is essential for designing integrated care pathways and informing personalised medicine. Chronic and complex diseases such as cancer, diabetes, and cardiovascular disorders often evolve dynamically, with biomarkers and clinical events influencing each other over time. For example, in oncology, tumor markers and radiological progression interact bidirectionally with overall survival [8]. In diabetes, glycemic trajectories both predict and are altered by the occurrence of cardiovascular events [7]. Similarly, in liver transplantation, graft function biomarkers evolve with complications and can in turn be accelerated by post-transplant events. Such interdependencies underscore the need for statistical frameworks capable of handling feedback loops between longitudinal and survival processes. Traditional joint modeling approaches, originally introduced by [9], provide a coherent likelihood-based framework for linking longitudinal biomarkers with time-to-event outcomes. These models have been widely extended to incorporate flexible association structures [10], dynamic prediction methods [8], and more recently, computational strategies for large-scale electronic health record (EHR) data through the jmBIG package [11]. However, two major challenges remain. First, existing methods largely assume a unidirectional relationship, where biomarkers influence events but not vice versa. Second, clinical uptake is limited by the lack of accessible, user-friendly tools for applied researchers and clinicians to explore and interpret such models in real time.

### Positioning relative to existing frameworks

Several established software packages implement joint models: JMbayes2 [12] provides Bayesian estimation with flexible association structures (current value, slope, cumulative effects) but assumes unidirectional biomarker-to-event influence; joineRML [13] offers multivariate random-effects joint models via Monte Carlo EM but does not accommodate semi-parametric links; FastJM [14] provides computationally efficient cause-specific hazard models but restricts the association to linear functional forms; and rstanarm [15] supports Bayesian joint models with Stan but lacks explicit feedback mechanisms. The semi-parametric association structures in the present work are related to the penalized spline approaches of [16] for flexible covariate effects in survival models and the neural additive model framework of [17], but neither of these has been integrated within a joint longitudinal–survival model with bidirectional feedback. Multi-state models [18, 19] offer an alternative approach to modeling transitions between disease states, but they do not directly link continuous biomarker trajectories to transition intensities nor accommodate the semi-parametric functional forms we consider. More recently, deep-learning–based dynamic survival models such as Dynamic-DeepHit [20] and recurrent-network joint models [21] have been proposed for very large EHR cohorts; these methods scale well but typically sacrifice the explicit, interpretable feedback structure and pointwise uncertainty quantification that our framework retains. Functional joint models that treat the longitudinal process as a smooth function [22] are another complementary direction, but they do not formalize event-induced trajectory discontinuities. Our contribution thus lies at the intersection of flexible association modeling, explicit feedback encoding, and joint longitudinal–survival inference—a combination not addressed by any single existing framework.

To address these gaps, we introduce JMbdirect, an R package and interactive dashboard designed to support joint modeling of longitudinal and survival data with explicit incorporation of bidirectional feedback. Unlike jmBIG, which emphasizes scalability to millions of observations, JMbdirect focuses on methodological innovation (semi-parametric link functions, neural additive components, penalized splines) and practical usability for translational research. The dashboard (https://atanu.shinyapps.io/jmbdirectapp/) enables users to upload datasets, fit joint models, visualize biomarker-event relationships, and generate personalized dynamic predictions without requiring advanced coding skills. This functionality directly addresses the growing demand for personalized time-to-event risk prediction, where clinicians need tools that can continuously update prognosis as new biomarker information becomes available.

### Bidirectional interdependencies in chronic diseases

Chronic diseases such as cardiovascular disease, type 2 diabetes, chronic kidney disease (CKD), and cancer are marked by strong bidirectional interdependencies, driven by shared risk factors, overlapping pathophysiologies, and cumulative burden over time. For example, diabetes increases risk of both cardiovascular disease and CKD through mechanisms including hyperglycemia, systemic inflammation, and endothelial dysfunction; in turn, the onset of CKD exacerbates glucose dysregulation and accelerates cardiovascular deterioration [23]. Similarly, in oncology, recurrent events alter inflammatory and immune biomarker trajectories, which then modify risks of further progression or mortality.

At the mechanistic level, pathways such as chronic inflammation, oxidative stress, insulin resistance, mitochondrial dysfunction, and immune dysregulation link these multimorbid conditions. Pro-inflammatory cytokines (e.g., IL-6, TNF-*α*) contribute to insulin resistance and carcinogenesis, while advanced glycation end-products (AGEs) accelerate vascular and renal damage [24–26]. These interactions highlight the necessity of statistical models capable of capturing not just unidirectional associations but genuine bidirectional feedback dynamics. In particular, the biological evidence for cross-disease reinforcement—where events in one disease domain accelerate deterioration in another—motivates the statistical formalization of feedback mechanisms that we develop in Methods section [27, 28].

### The need for dynamic, personalized risk prediction

Static prediction tools, such as baseline risk scores, are limited because they cannot adapt to the evolving interplay between biomarkers and clinical events. In contrast, dynamic joint modeling allows real-time risk updating as new longitudinal information accumulates [5, 29]. This is particularly important in multimorbid patients, where deterioration in one domain (e.g., renal function) may signal accelerated risk in another (e.g., cardiovascular events). Evidence from clinical studies in diabetes, oncology, and transplantation demonstrates that dynamic prediction identifies high-risk patients earlier and supports personalized intervention strategies [29–31].

To facilitate translation of these methods into practice, we have developed interactive dashboards that integrate model-based risk predictions with real-time visualization of biomarker trajectories and survival outcomes. These tools bridge the gap between advanced modeling frameworks and clinical usability, supporting both decision-making and communication between clinicians and patients.

### Methodological advances with JMbdirect

Building upon prior scalable approaches such as the jmBIG package for large-scale EHR data [11], the present work introduces JMbdirect, a methodological extension designed specifically for bidirectional joint modeling with semi-parametric link functions. Unlike jmBIG, which primarily addresses scalability, JMbdirect focuses on methodological innovation: (i) modeling bidirectional feedback between longitudinal biomarkers and events, (ii) incorporating semi-parametric link functions to flexibly capture non-linear associations, and (iii) supporting realistic clinical prediction tasks where both biomarkers and event processes evolve interactively.

A natural question is why JMbdirect was not built as an extension of jmBIG. The reason is architectural: jmBIG is optimized for scalability through data partitioning and parallelized estimation on large EHR cohorts, whereas the semi-parametric link functions and bidirectional feedback structures introduced here require fundamentally different estimation routines (penalized splines within the M-step, neural additive component optimization, and feedback-augmented trajectory updates). Embedding these within jmBIG’s partitioned-data architecture would introduce prohibitive coupling between partitions, since the feedback terms and flexible association functions require subject-level trajectory information that cannot be easily decomposed across data shards. Nevertheless, the JMbdirect dashboard does provide a jmBIG pathway for users who need to scale estimation to larger cohorts, using a modular interface that routes to the appropriate backend depending on dataset size and modeling requirements.

In oncology, for instance, locoregional recurrence (LRC) not only reflects tumor aggressiveness but also alters trajectories of immune and inflammatory biomarkers, which then influence progression-free survival (PFS) and overall survival (OS). In chronic disease management, a cardiovascular event may permanently shift glycemic trajectories in diabetes patients, necessitating more aggressive treatment strategies. Capturing such feedback loops is central to JMbdirect.

This paper makes three distinct contributions: (i) methodological advances in joint modeling through semi-parametric association structures and bidirectional feedback formulations, (ii) implementation of these advances in the open-source R package JMbdirect, and (iii) development of an interactive dashboard for clinical application that makes these methods accessible to applied researchers and clinicians without requiring advanced coding skills. The primary focus is methodological: we develop, formalize, and validate the statistical framework, while the software and dashboard serve as vehicles for translating this methodology into practice.

### Challenges and rationale

**Fig. 1:**
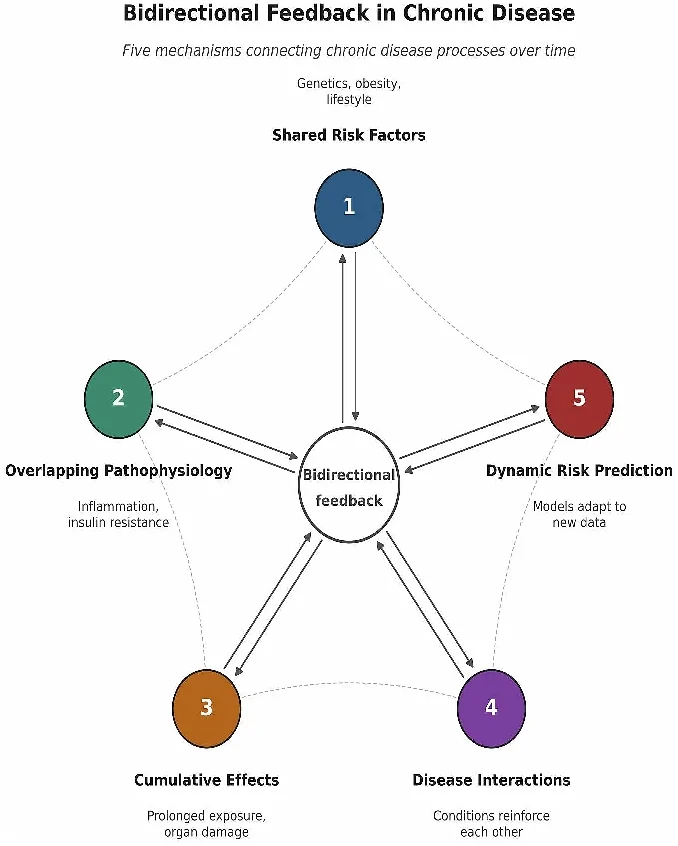
Conceptual framework illustrating bidirectional relationships among chronic diseases, including shared risk factors (e.g., inflammation), overlapping pathophysiologies, disease interactions, cumulative effects, and the role of dynamic risk prediction in creating feedback loops

Several challenges remain in operationalizing bidirectional joint models. Data quality issues such as sparsity, missingness, and temporal misalignment are pervasive in EHR-based studies [32]. Multimodal integration of clinical, genomic, and imaging data requires harmonization and scalable inference. Furthermore, interpretability and transparency are critical to clinician adoption, emphasizing the need for visual dashboards and intuitive outputs. By combining flexible semi-parametric link functions, bidirectional modeling, and interactive implementation, JMbdirect addresses these challenges while aligning with the goals of precision medicine. Its development reflects a methodological and translational advance beyond existing frameworks, enabling clinically meaningful dynamic risk prediction in multimorbid populations (Fig. 1).

## Methods

This section presents the full mathematical framework, the software and dashboard implementation, and the design of the simulation study used to evaluate the proposed methods. To aid the reader, we note that the core longitudinal and survival submodels are first stated compactly in Bidirectional extension of joint models–Semi-parametric association structures sections to motivate the bidirectional extension, and then elaborated with estimation details in Relevance to multimorbidity prediction–Software implementation: JMbdirect sections. Where equations reappear (e.g., the longitudinal submodel), this is intentional to maintain self-containedness of each subsection; we have minimized redundancy where possible.

### Overview of the joint modeling framework

Joint models of longitudinal and survival data provide a principled approach for analyzing the interplay between evolving biomarkers and time-to-event processes [5, 9, 10, 33]. Classical formulations impose a unidirectional flow: the longitudinal process drives the risk of event occurrence, while the event does not subsequently influence the biomarker trajectory. This assumption, though convenient, is inadequate in realistic clinical scenarios. For example, relapse in cancer typically induces sharp declines in quality-of-life scores, while hospitalizations in heart failure abruptly alter trajectories of cardiac biomarkers. Failure to accommodate this feedback risks biased estimation and poor predictive calibration.

### Bidirectional extension of joint models

We propose a bidirectional extension, implemented in the JMbdirect framework, that explicitly encodes feedback from events into longitudinal trajectories. For subject *i=1,… ,n*, the longitudinal submodel is

1 $$\begin{aligned} Y_i(t) = X_i(t)^{\top }\beta + Z_i(t)^{\top }b_i + \epsilon _i(t), \quad \epsilon _i(t)\sim \mathcal {N}(0,\sigma ^2), \end{aligned}$$

with $b_i \sim \mathcal {N}(0,D)$ capturing subject-specific heterogeneity. Here $Y_i(t) \in \mathbb {R}$ is the observed longitudinal biomarker for subject *i* at time *t*; $X_i(t) \in \mathbb {R}^p$ is the design vector for the population fixed effects *β* (typically a baseline, treatment indicator, time *t*, and treatment-by-time interaction); $Z_i(t) \in \mathbb {R}^q$ is the design vector for the subject-specific random effects $b_i \in \mathbb {R}^q$ (typically a random intercept and a random slope on time); $\varepsilon _i(t) \sim \mathcal {N}(0, \sigma ^2)$ is independent within-subject Gaussian measurement error; and $b_i \sim \mathcal {N}(0, D)$ with *D* a *q × q* positive-definite covariance matrix. The fitted smooth trajectory is

2 $$\begin{aligned} m_i(t) = X_i(t)^{\top }\beta + Z_i(t)^{\top }b_i. \end{aligned}$$

The survival component for competing event types *k=1,… ,K* is specified by

3 $$\begin{aligned} h_{ik}(t) = h_{0k}(t)\exp \!\left( W_i^{\top }\gamma _k + f_k(m_i(t)) \right) , \end{aligned}$$

where $h_{0k}(t)$ is a baseline hazard, $W_i$ are baseline covariates, $\gamma _k$ are regression parameters, and $f_k(\cdot )$ is a semi-parametric association function.

We note that when $f_k(\cdot )$ is non-linear, the interpretation of the biomarker–hazard association is no longer reducible to a single hazard ratio per unit change in $m_i(t)$. Instead, the instantaneous effect of the biomarker on the log-hazard is given by the derivative $f_k'(m_i(t))$, which varies across the biomarker range. This provides richer clinical interpretation—for example, identifying threshold values beyond which risk escalates sharply—but requires visualization of the estimated $f_k(\cdot )$ curve (and its pointwise confidence/credible band) rather than a single summary parameter. The dashboard provides such visualizations to support interpretability.

Our use of cause-specific hazards in Eq. (3) requires that censoring by the competing event is non-informative *conditional on the model*, including the feedback-augmented trajectory. Concretely, given $b_i$ and the time-indexed event filtration $\mathcal {H}_i^E(t)$, the censoring time $C_i$ and the latent event times for the other causes are assumed conditionally independent. The bidirectional feedback structure does not weaken this assumption: although an event in cause 1 changes the biomarker (and hence the hazard for cause 2), this dependence is captured by the model itself rather than relegated to the censoring mechanism. Investigators should still verify the plausibility of conditional independence in applied settings—for example, by checking whether administrative censoring patterns differ systematically across treatment groups or biomarker strata.

To capture feedback, the trajectory is augmented with post-event dynamics:

4 $$\begin{aligned} m_i(t) = X_i(t)^{\top }\beta + Z_i(t)^{\top }b_i + \sum \limits _{j \in \mathcal {J}_i(t)} \left\{ \kappa _{j0}\, \mathbb {I}(t \ge T_{ij}) + \kappa _{j1}\,(t - T_{ij})_+ \right\} , \end{aligned}$$

where $T_{ij}$ are non-terminal intermediate event times (e.g., non-fatal recurrence, hospitalisation, decompensation episode), $(\cdot )_+=\max (0,\cdot )$ denotes the truncated linear basis, and $\kappa _j = (\kappa _{j0}, \kappa _{j1})^\top$ collects the post-event level shift and slope change. The index set $\mathcal {J}_i(t) = \{ j : T_{ij} \le t,\, T_{ij} \le C_i,\, T_{ij} \text { is non-terminal} \}$ is time- and subject-specific, containing only intermediate events that have already occurred for subject *i* by time *t*. The feedback augmentation applies only to such non-terminal events. For a terminal event (e.g., death, or transplant when transplant is itself the endpoint of interest), the longitudinal process $Y_i(t)$ is undefined for $t > T_i^{\textrm{terminal}}$, no post-terminal-event measurements are generated, and no feedback term contributes to the likelihood beyond $\min (t, T_i^{\textrm{terminal}}, C_i)$. This formulation permits abrupt level shifts and slope changes after non-terminal events, thereby encoding reciprocal causality between disease progression and events while respecting the natural causal ordering.

#### Event-specific versus shared *κ*

In principle, Eq. (4) writes $\kappa _j$ as event-specific, but identifiability requires care. We adopt the following hierarchy by default. Feedback parameters are shared across all non-terminal events of the *same type* (e.g., all decompensation episodes for a given subject and across subjects), yielding a small number of type-specific parameter pairs $(\kappa _{[\textrm{type}],0}, \kappa _{[\textrm{type}],1})$ that are estimated globally. This is the default in JMbdirect and the specification used throughout our simulations and the PBC application. Subject-specific *κ* values are typically unidentifiable when the number of post-event observations per subject is small and are not used in the default pipeline. When the number of intermediate event types is large or when type-specific effects are themselves heterogeneous, a random-effects extension $\kappa _{[\textrm{type}]} \sim \mathcal {N}(\kappa _{[\textrm{type}]}^\star , \Sigma _\kappa )$ can be specified, but this requires a sufficient number of subjects experiencing each event type (typically *≥ 30*).

#### Identifiability of *κ* when intermediate events are rare

The feedback parameters require post-event longitudinal observations to be identified. Specifically, $\kappa _{j0}$ (level shift) requires at least one post-event measurement; $\kappa _{j1}$ (slope change) requires at least two. In practice we recommend that no fewer than *≈ 5* post-event observations per event type be available across the cohort for stable estimation of the pair, and at least one such observation per subject experiencing the event for the subject-level contribution to be informative. When these conditions are violated, the M-step update for *κ* becomes numerically unstable and the penalised log-likelihood can fail to converge; the JMbdirect estimation routine in such cases issues a diagnostic warning and falls back to a level-only model ($\kappa _{j1} \equiv 0$) or, if even level effects are not identifiable, to the unidirectional joint model with $\kappa \equiv 0$. The dashboard surfaces this diagnostic to the user.

#### Tuning and estimation of *κ*

The feedback parameters $\kappa _j = (\kappa _{j0}, \kappa _{j1})^\top$ governing post-event level shifts and slope changes are not pre-specified but are estimated jointly with all other model parameters within the likelihood framework. In the penalized maximum likelihood approach, $\kappa _j$ are updated in the M-step alongside *β* and the spline/neural coefficients; in the Bayesian approach, weakly informative priors $\kappa _{j0} \sim \mathcal {N}(0, \sigma ^2_\kappa )$ and $\kappa _{j1} \sim \mathcal {N}(0, \sigma ^2_\kappa )$ are assigned, with $\sigma ^2_\kappa$ chosen to reflect clinically plausible magnitudes of post-event biomarker shifts (e.g., for bilirubin, a prior standard deviation of 2–3 mg/dL). Profile likelihood analyses or posterior summaries of $\kappa _j$ quantify the magnitude and uncertainty of event-induced trajectory changes. In Results section, we present sensitivity analyses examining how different true values of *κ* affect estimation accuracy and predictive performance, confirming that moderate-to-large feedback effects are reliably recovered while small effects may require larger sample sizes for precise estimation. Both components have direct clinical interpretation. $\kappa _{j0}$ is the instantaneous shift in the biomarker level immediately after a non-terminal event, expressed on the same scale as $Y_i(t)$; $\kappa _{j1}$ is the post-event change in the rate of biomarker evolution per unit of time. Clinically, *κ* encodes “shock” and “trend-break” effects that unidirectional joint models cannot capture and that would otherwise be absorbed (incorrectly) into baseline random-effects variability. Indicative magnitudes in our applications are: for PBC, $\kappa _{j0} \approx 0.5$ (log mg/dL) corresponds to the bilirubin elevation observed after a decompensation episode, with $\kappa _{j1} \approx 0.10$ capturing the accompanying acceleration in decline; for oncology applications, $\kappa _{j0}$ in the range 1.0–1.5 on the log scale is consistent with abrupt CA-125 elevations following radiologically-confirmed ovarian-cancer recurrence; and in nephrology, $\kappa _{j1}$ captures the well-documented acceleration in eGFR decline following a first cardiovascular hospitalisation.

### Semi-parametric association structures

Classical joint models rely on linear functional forms linking longitudinal processes with event risks, which can obscure nonlinear phenomena such as biomarker thresholds or saturation effects. To overcome this limitation, we extend the association structure by introducing semi-parametric components $f_k(\cdot )$ that allow the data to dictate functional shape rather than pre-imposing linearity.

Formally, we specify

5 $$\begin{aligned} f_k(m) = \sum \limits _{\ell =1}^{L} \alpha _{\ell } B_{\ell }(m), \end{aligned}$$

where $B_{\ell }(m)$ are B-spline basis functions and smoothness is controlled via a roughness penalty on adjacent coefficients. This penalized spline representation ensures flexibility while avoiding overfitting. An alternative specification adopts neural additive architectures,

6 $$\begin{aligned} f_k(m) = \sum \limits _{q=1}^Q \sigma (a_q m + b_q), \end{aligned}$$

with $\sigma (\cdot )$ an activation function. Regularization through $L_1/L_2$ penalties constrains the effective complexity, enabling the model to approximate threshold-like responses or nonlinear amplification effects. These formulations provide a methodological bridge between statistical spline models and machine-learning–oriented functional approximators, enhancing the capacity of joint models to capture multimorbidity-driven nonlinearities.

#### Note on B-spline non-orthogonality

The B-spline basis is not orthogonal, so the individual coefficients $\alpha _\ell$ are not separately interpretable; inference and reporting are therefore conducted on the fitted curve $\hat{f}_k(m)$ and its pointwise confidence/credible band rather than on the coefficient scale. The second-order difference penalty in the M-step shrinks adjacent coefficients toward smoothness, which dramatically reduces the effective degrees of freedom and stabilises the inversion of the penalised information matrix. We additionally standardise $m_i(t)$ before basis evaluation and place knots at empirical deciles of the predicted $m_i(t)$ distribution rather than at fixed equispaced locations, which keeps the basis well-conditioned across realistic biomarker ranges. Sensitivity analyses reported in Results section (knot count from 5 to 15; alternative truncated-power and natural-cubic-spline bases) confirm that the methodological inference is robust to the specific non-orthogonal parameterisation.

### Estimation strategy

The resulting likelihood combines contributions from longitudinal and event processes, marginalized over unobserved random effects:

7 $$\begin{aligned} L(\theta ) = \prod _{i=1}^n \int p(Y_i|b_i;\theta ) \, p(T_i,\delta _i|b_i;\theta ) \, p(b_i;\theta ) \, db_i, \end{aligned}$$

where $\delta _i$ denotes the competing risk indicator. The parameter vector *θ* collects all unknown quantities of the model: the longitudinal fixed effects *β*; the measurement-error variance $\sigma ^2$; the random-effects covariance *D* (parameterised by its unique entries $\textrm{vech}(D)$); the cause-specific baseline-hazard parameters $\{h_{0k}(\cdot )\}_{k=1}^K$ (either Weibull shape and scale, or piecewise-constant log-rates on a chosen partition); the cause-specific covariate effects $\{\gamma _k\}_{k=1}^K$; the semi-parametric link parameters (spline coefficients $\{\alpha _\ell \}_{\ell =1}^L$ or NAM weights $\{a_q, b_q\}_{q=1}^Q$); and the feedback parameters $\{\kappa _j\}$. A consolidated notation table is provided at the end of this subsection. Since the association functions $f_k(\cdot )$ no longer admit closed-form likelihood components, standard numerical integration schemes become unstable or computationally prohibitive.

#### Conditional independence under feedback

We acknowledge that the augmentation of trajectories with the post-event terms in Eq. (4) introduces a dependence between the longitudinal observations $Y_i(t)$ for $t > T_{ij}$ and the past event history, which technically violates the conditional independence assumption $p(Y_i, T_i | b_i) = p(Y_i | b_i) \, p(T_i | b_i)$ that underpins the factored likelihood in classical joint models. In our framework, this dependence is respected by conditioning the longitudinal density on the *time-indexed* event filtration $\mathcal {H}_i^E(t) = \{ T_{ij} : T_{ij} \le t,\, T_{ij} \le C_i \}$, which contains only events that have already occurred by time *t* and therefore preserves the causal ordering (the longitudinal process at *t* cannot depend on events that have not yet occurred). The likelihood is constructed as

8 $$\begin{aligned} L_i(\theta ) = \int \left[ \prod _{t \in \mathcal {T}_i} p\!\left( Y_i(t) \mid b_i, \mathcal {H}_i^E(t); \theta \right) \right] \, p\!\left( \mathcal {H}_i^E(C_i) \mid b_i; \theta \right) \, p(b_i; \theta ) \, db_i, \end{aligned}$$

where $\mathcal {T}_i$ denotes the set of observation times for subject *i*. The longitudinal contribution at each *t* is conditioned only on events in $\mathcal {H}_i^E(t)$ — not on future events — so the augmentation in Eq. (4) does not introduce anticipation. The survival component $p(\mathcal {H}_i^E(C_i) \mid b_i; \theta )$ remains specified through cause-specific hazards as in Eq. (3). This formulation preserves the coherence of the joint likelihood while explicitly accommodating the feedback structure.

#### Treatment of the visit/observation process in estimation

The main results in Results section assume that the visit process is non-informative conditional on the model, i.e. that the timing of biomarker measurements does not depend on the latent biomarker state beyond what is captured by observed covariates. The data-generating simulations in Informative dropout and observation process section deliberately violate this assumption (visit intensity depends on $m_i(t)$) to quantify the resulting bias. As reported in the Discussion, modest violations of non-informative observation produce only mild attenuation of estimates in our simulations; severe violations would require joint modeling of the visit-intensity process, which is supported in the JMbdirect estimation routine through an optional intensity submodel but is not invoked by default.

To address this, we develop two complementary estimation strategies:

1. **Penalized maximum likelihood**. An EM-type algorithm is constructed with Laplace approximation to evaluate the random-effects integrals, while the spline/neural components are estimated under penalty constraints. This extension generalizes existing EM implementations by incorporating semi-parametric penalties within the M-step.
2. **Bayesian inference**. A hierarchical model is specified with priors on spline coefficients or neural weights, ensuring automatic smoothness selection. Posterior sampling is carried out via Hamiltonian Monte Carlo implemented in Stan, allowing full uncertainty quantification and facilitating model comparison through marginal likelihood and predictive checks.

#### Detailed penalized EM algorithm

We provide a more explicit description of the penalized maximum likelihood procedure, which proceeds iteratively as follows.

*E-step.* Given current parameter estimates $\theta ^{(r)}$, the posterior distribution of random effects for each subject is approximated via the Laplace method:

$$\begin{aligned} \hat{b}_i = \arg \max _{b_i} \left\{ \log p(Y_i | b_i; \theta ^{(r)}) + \log p(\mathcal {H}_i^E(C_i) | b_i; \theta ^{(r)}) + \log p(b_i; \theta ^{(r)}) \right\} , \end{aligned}$$

with the posterior covariance obtained from the negative inverse Hessian at $\hat{b}_i$. This yields approximate conditional expectations $\mathbb {E}[g(b_i) | Y_i, \mathcal {H}_i^E(C_i); \theta ^{(r)}]$ for the sufficient statistics needed in the M-step.

*M-step (longitudinal parameters).* Fixed effects *β* and variance components $(\sigma ^2, D)$ are updated by maximizing the expected complete-data log-likelihood, which reduces to weighted least squares for *β* and restricted maximum likelihood updates for $\sigma ^2$ and *D*.

*M-step (feedback parameters).* The feedback coefficients $\kappa _j$ are updated jointly with *β* by including the post-event basis functions $(t - T_{ij})_+$ and $\mathbb {I}(t \ge T_{ij})$ in the design matrix. No additional penalty is imposed on $\kappa _j$; they are estimated as standard regression coefficients conditional on the event history.

*M-step (survival parameters and semi-parametric link).* The baseline hazard parameters, covariate effects $\gamma _k$, and spline coefficients $\alpha _\ell$ (or neural weights $a_q, b_q$) are updated by maximizing the penalized expected log-partial-likelihood:

$$\begin{aligned} \ell _{\textrm{pen}}(\gamma _k, \alpha , \lambda ) = \sum \limits _{i=1}^n \mathbb {E}\!\left[ \log p(\mathcal {H}_i^E(C_i) | b_i; \theta ) \,\left| \, Y_i, \mathcal {H}_i^E(C_i); \theta ^{(r)}\right. \right] - \lambda \sum \limits _{\ell =2}^L (\Delta ^2 \alpha _\ell )^2, \end{aligned}$$

where $\Delta ^2 \alpha _\ell = \alpha _{\ell } - 2\alpha _{\ell -1} + \alpha _{\ell -2}$ is the second-order difference penalty and *λ* is the smoothing parameter selected by GCV at each iteration. For NAM specifications, the M-step is modified as follows.

*M-step under the NAM specification.* When $f_k(\cdot )$ is given by Eq. (6) rather than Eq. (5), the penalty term $-\lambda \sum _\ell (\Delta ^2 \alpha _\ell )^2$ is replaced by an elastic-net penalty on the NAM parameters,

$$\begin{aligned} - \lambda _1 \sum \limits _{q=1}^{Q} |a_q| - \lambda _2 \sum \limits _{q=1}^{Q} \left( a_q^2 + b_q^2\right) , \end{aligned}$$

and the survival-parameter update is performed by mini-batch stochastic gradient ascent on $\ell _{\textrm{pen}}$ using the Adam optimiser (learning rate $10^{-3}$, $\beta _1 = 0.9$, $\beta _2 = 0.999$). Mini-batches are formed at the subject level (not the observation level) so that within-subject correlation is preserved. The penalty hyperparameters $(\lambda _1, \lambda _2)$ are selected by 5-fold cross-validation on the integrated Brier score, refit at each outer EM iteration. Pointwise uncertainty bands for $\hat{f}_k(m)$ under the NAM specification are obtained by parametric bootstrap (500 resamples of the random-effects posterior with NAM-head refits), in contrast to the spline case where bands are available analytically from the penalised information matrix.

*Convergence.* The algorithm iterates until the relative change in penalized log-likelihood falls below a tolerance (default $10^{-5}$) or a maximum number of iterations (default 200) is reached. Standard errors are obtained from the observed information matrix evaluated at convergence, with the penalty term included to yield appropriately inflated variances for the spline coefficients.

*Bayesian estimation details.* For the Bayesian approach, the hierarchical model specifies: $\beta \sim \mathcal {N}(0, 100 I)$; $\sigma ^2 \sim \text {Inv-Gamma}(0.01, 0.01)$; $D \sim \text {Inv-Wishart}(\nu _0, \Psi _0)$ with $\nu _0 = q + 1$ and $\Psi _0 = I$; spline coefficients $\alpha _\ell \sim \mathcal {N}(0, \tau ^2)$ with $\tau ^2 \sim \text {Half-Cauchy}(0, 1)$ providing automatic smoothness selection; and $\kappa _j \sim \mathcal {N}(0, \sigma ^2_\kappa )$ as described in Bidirectional extension of joint models section. Hamiltonian Monte Carlo sampling uses 4 chains of 2000 iterations each (1000 warmup), with convergence assessed via $\hat{R} < 1.01$ and effective sample sizes exceeding 400 per parameter.

#### Dynamic prediction

For dynamic prediction of cause-*m* survival beyond a landmark time *t*, given the observed biomarker and event history $\mathcal {D}_i(t) = \{Y_i(s): s \le t\} \cup \{\mathcal {H}_i^E(t)\}$, the target functional is

9 $$\begin{aligned} \pi _{im}(u \mid t) &= \Pr (T_{im}> u \mid T_i> t, \mathcal {D}_i(t); \theta ) \\&= \int \Pr (T_{im}> u \mid T_i > t, b_i; \theta )\, p(b_i \mid \mathcal {D}_i(t); \theta )\, db_i, \end{aligned}$$

following the Monte Carlo scheme of [29]. The posterior $p(b_i \mid \mathcal {D}_i(t); \theta )$ is approximated by Laplace at $\hat{b}_i(t)$ (frequentist) or by direct posterior sampling (Bayesian). The inner conditional survival is evaluated by numerical integration of the cause-specific hazard in Eq. (3) over [*t*, *u*], taking care to project the feedback-augmented trajectory $m_i(s)$ forward using the events realized in $\mathcal {H}_i^E(t)$ but not anticipating events after *t*. Predictions are summarized by their posterior mean and 95% credible (or confidence) interval. We emphasize that in the presence of competing risks, cause-specific survival as in Eq. (9) is distinct from the cumulative incidence function (see JMbayes2 module for two events (jmbB) section).

#### Choice between estimation paradigms

The decision between penalized maximum likelihood and Bayesian inference should be guided primarily by the scientific context rather than computational convenience alone. When substantive prior knowledge is available—for example, clinically established ranges for biomarker effects, known directions of feedback, or informative historical data—the Bayesian framework is preferred because it formally incorporates this knowledge and yields posterior distributions that directly support clinical interpretation through credible intervals and posterior predictive checks. The penalized likelihood approach is appropriate when prior information is limited or when the primary goal is scalable estimation in large datasets where full MCMC sampling becomes prohibitive. In practice, we recommend fitting both when feasible, using the Bayesian analysis as the primary inferential tool and the penalized likelihood fit as a computational cross-check.

These estimation strategies are not merely computational alternatives but methodological complements: the penalized likelihood approach provides efficient inference when prior information is sparse or when dataset size makes MCMC impractical, while the Bayesian formulation yields richer posterior summaries and facilitates principled incorporation of prior knowledge.

#### Notation

Table 1 summarises the notation used throughout the Methods.

**Table 1:** Notation used in the methodological framework

Symbol	Meaning
*i = 1, … , n*	Subject index
*k = 1, … , K*	Cause-specific event index (competing risks)
*t*	Continuous time since baseline
$Y_i(t)$	Observed longitudinal biomarker for subject *i* at time *t*
$m_i(t)$	Underlying smooth trajectory (free of measurement error)
$X_i(t), \beta$	Fixed-effects design vector and parameter vector for $Y_i(t)$
$Z_i(t), b_i$	Random-effects design vector and subject-specific random effects
$\varepsilon _i(t), \sigma ^2$	Within-subject Gaussian measurement error and its variance
*D*	Covariance matrix of $b_i$
$W_i, \gamma _k$	Baseline covariates and cause-specific covariate effects
$h_{0k}(t)$	Cause-specific baseline hazard for event type *k*
$h_{ik}(t)$	Cause-specific hazard for subject *i* and event type *k*
$f_k(\cdot )$	Semi-parametric association function for cause *k*
$B_\ell (\cdot ), \alpha _\ell$	B-spline basis functions and spline coefficients
$a_q, b_q$	Neural additive component weights and offsets
$T_{ij}$	Time of the *j*-th non-terminal event for subject *i*
$\mathcal {J}_i(t)$	Set of non-terminal events that have occurred by time *t*
$\kappa _{j0}, \kappa _{j1}$	Post-event level shift and slope change after event *j*
$T_i^{\textrm{terminal}}, C_i$	Terminal event time and administrative censoring time
$\mathcal {H}_i^E(t)$	Time-indexed event filtration at time *t*
$\delta _i$	Cause-specific event indicator
$\pi _{im}(u|t)$	Dynamic prediction of cause-*m* survival at horizon *u* given history through *t*
$\mathcal {D}_i(t)$	Observed biomarker and event history through time *t*
*θ*	Full parameter vector
*λ*	Smoothing parameter for the spline penalty (selected by GCV)

### Methodological contribution

The proposed framework constitutes a methodological extension of classical joint models along three key dimensions:

1. **Flexible association structures.** By replacing fixed linear terms with semi-parametric functions (splines or neural additive components), the model can uncover nonlinear patterns such as biomarker thresholds, saturation plateaus, and interaction effects across multimorbidity pathways. This bridges the gap between traditional joint models and contemporary machine learning approaches.
2. **Bidirectional integration.** The association functions are embedded within a structure that allows feedback between event processes and longitudinal trajectories. This contrasts with the unidirectional specification of conventional joint models and reflects more realistic clinical dynamics where events (e.g., recurrence, hospitalization) alter biomarker evolution.
3. **Hybrid estimation schemes.** The use of penalized maximum likelihood with Laplace approximation provides scalability for high-dimensional data, while Bayesian inference with hierarchical priors enables richer uncertainty quantification and principled incorporation of prior biomedical knowledge. The dual perspective ensures methodological robustness across settings with different computational or inferential demands.

Taken together, these extensions deliver a unified modeling framework capable of capturing nonlinear, feedback-driven, and multimorbidity-sensitive dynamics. Beyond offering greater predictive accuracy, the methodology enhances interpretability by revealing clinically meaningful thresholds and trajectories, while retaining theoretical rigor in estimation and inference.

### Relevance to multimorbidity prediction

Multimorbidity represents a system of interdependent disease processes where trajectories and risks evolve jointly rather than independently [3, 4]. Conventional unidirectional joint models, which assume conditional independence of morbidities given covariates, often understate cumulative risks and miscalibrate survival predictions. Our bidirectional, semi-parametric formulation addresses this limitation by allowing reciprocal pathways of influence between longitudinal processes and events, thereby aligning statistical inference with the biological interdependence of chronic diseases.

Formally, let $(Y_{i1}(t), \dots , Y_{iM}(t))$ denote *M* longitudinal biomarkers representing distinct morbidities, and $(T_{i1}, \dots , T_{iM})$ the corresponding event times. The hazard for morbidity *m* is modeled as

10 $$\begin{aligned} h_{im}(t) = h_{0m}(t)\exp \!\left( W_i^{\top }\gamma _m + f_m\left( m_{i1}(t),\dots ,m_{iM}(t)\right) \right) , \end{aligned}$$

so that the instantaneous risk of one condition depends on the evolving states of the others. Furthermore, events $T_{ij}$ induce discontinuities or accelerations in trajectories $m_{ik}(t)$, *k ≠ j*, capturing cross-disease spillover effects such as post-stroke renal decline or cancer recurrence–driven deterioration of systemic markers. This contrasts with additive independent hazards, and moves toward a network-based representation of multimorbidity.

The individualized, time-updating survival probability is the dynamic prediction $\pi _{im}(u \mid t)$ defined in Eq. (9), now generalised across the *M* morbidities. The backward feedback mechanism ensures that predictions incorporate distortions induced by earlier events, a feature absent from classical frameworks. This duality is essential in multimorbidity, where neglecting reciprocal influences has been shown to yield systematically biased risk estimates [7].

In sum, the methodological contribution lies in extending joint modeling from a disease-centric paradigm to a system-level, feedback-sensitive formalism. This provides a mathematically rigorous and practically interpretable basis for multimorbidity risk prediction, supporting clinical decision-making in complex patient populations.

### Dashboard implementation

An interactive dashboard has been developed and is publicly accessible at https://atanu.shinyapps.io/jmbdirectapp/. The application provides an integrated environment where advanced bidirectional joint models can be fitted without extensive coding. Users may upload their own longitudinal and survival datasets or explore reference cohorts, after which the system automatically estimates trajectories, event risks, and individualized dynamic predictions.

#### Data privacy and de-identification

When using the dashboard with real patient data, users must ensure that all uploaded datasets have been fully de-identified in accordance with applicable privacy regulations (e.g., HIPAA in the United States, GDPR in the European Union). The dashboard does not store uploaded data persistently and processes all analyses in ephemeral server sessions; however, the hosting platform and the authors cannot guarantee the security of data transmitted over the internet. A prominent disclaimer to this effect is displayed on the dashboard landing page, and users are advised to remove all direct identifiers (names, dates of birth, medical record numbers, etc.) and to apply appropriate de-identification procedures before upload. The manuscript authors bear no responsibility for breaches arising from the upload of identifiable patient data.

The dashboard has been designed with translational applications in mind. In oncology, users can link tumor marker trajectories with local control, progression, and overall survival to support individualized trial monitoring and patient counselling. In nephrology, declining eGFR trajectories can be combined with endpoints such as dialysis initiation and mortality, providing forward-looking estimates that are crucial for planning renal replacement therapy. In cardiometabolic multimorbidity, HbA1c and lipid dynamics can be jointly analyzed with cardiovascular and renal outcomes, highlighting reciprocal risk pathways.

For each scenario, the dashboard produces outputs including survival curves with prediction intervals, biomarker–event association plots, and patient-level dynamic risk profiles. These results can be updated in real time as new data are added, making the tool directly relevant for both trial settings and clinical decision support. By embedding methodological innovation in a practical interface, the platform bridges statistical development with real-world usability across multiple disease areas.

#### Visualization of bidirectional feedback

To aid interpretation of feedback effects, the dashboard includes dedicated visualization panels. These display: (i) estimated post-event trajectory shifts overlaid on pre-event biomarker projections, allowing users to visually assess the magnitude and direction of event-induced changes (governed by $\hat{\kappa }$); (ii) counterfactual trajectory comparisons showing predicted biomarker evolution with and without the occurrence of an event; and (iii) estimated $f_k(\cdot )$ association curves with pointwise uncertainty bands, revealing non-linear biomarker–hazard relationships. Together, these plots provide an intuitive, graphical means of learning about and communicating bidirectional feedback to both statistical and clinical audiences.

### Consolidated submodel specifications

The longitudinal submodel (Eq. 1), survival submodel with semi-parametric link (Eq. 3), semi-parametric link function families (Eqs. 5–6), bidirectional feedback formulation (Eq. 4), and estimation strategy (Eq. 7 and the detailed algorithm in Estimation strategy section) have been presented in full in Bidirectional extension of joint models–Estimation strategy sections above. To avoid redundancy, we do not restate them here but refer the reader to those sections for the complete mathematical specifications. The key modeling choices—linear mixed-effects structure for $Y_i(t)$, cause-specific hazards with flexible $f_k(\cdot )$, post-event trajectory modification via $\kappa _j$, and dual penalized-ML/Bayesian estimation—are unified in the JMbdirect software described next.

### Software implementation: JMbdirect

The entire framework is implemented in the R package JMbdirect [34], which enables direct modeling of bidirectional longitudinal–survival associations. Key functions such as jmb_direct() allow users to specify longitudinal and event submodels with flexible link functions. Output includes parameter estimates, dynamic risk predictions, and visualization tools for *f(· )*. To facilitate adoption in applied settings, we developed an interactive dashboard that integrates with JMbdirect, enabling clinicians and researchers to explore dynamic predictions in real time. This translational component bridges advanced methodology with bedside decision-making, addressing the pressing need for individualized risk prediction tools in modern precision medicine.

#### Software versions and reproducibility

All results in this manuscript were produced with JMbdirect version 0.4.0 (CRAN release), JMbayes2 0.5-0, joineRML 0.4.6, FastJM 1.4.2, rjags 4–15 with JAGS 4.3.2, and jmBIG 0.1.2, under R 4.4.1 on Ubuntu 22.04. Random seeds are recorded in each script header (the master seed is 20250101). Complete reproduction materials are archived on Zenodo (DOI to be assigned at publication) and mirror the public GitHub repository github.com/atanustat/JMbdirect; the repository is released under the MIT license.

#### Relationship to existing backends

JMbdirect builds on, rather than re-implements, several established backends. Specifically:

- **Two-event Bayesian (jmbB).** The jmbB() wrapper reuses JMbayes2’s MCMC engine for the linear-link portion of the model and its random-effects posterior sampling, then wraps our feedback-augmented design matrix and post-event basis around it. JMbayes2’s predict() methods for cumulative-incidence functions are reused for the dashboard’s predictions panel, transformed to survival probabilities as described in JMbayes2 module for two events (jmbB) section.
- **Cause-specific frequentist (JMCS).** We route the baseline cause-specific hazard estimation to FastJM and overlay our spline/NAM link externally.
- **Likelihood-based two-event (JMRML).** We reuse joineRML’s Monte Carlo EM for the linear-link multivariate joint model and use it as a triangulation check on our penalised-likelihood estimator.
- **Custom Bayesian feedback (JMSTJAGS).** The semi-parametric feedback model is sampled with rjags/JAGS using our own model files; this is the pathway where the full bidirectional semi-parametric specification is fit Bayesianly.
- **Scaling (jmBIG).** For large EHR cohorts the dashboard routes to jmBIG, leveraging its partitioned-data and parallelised-estimation architecture; the linkage is via lightweight prediction wrappers (e.g., predJMbayes) that mirror the small-sample interface.

Components that are *new* in JMbdirect (not present in any of the above backends) are: the semi-parametric link function families (Eqs. 5–6), the feedback augmentation of the trajectory (Eq. 4), the penalised EM updates for the feedback and link parameters (Estimation strategy section), and the dashboard integration.

### The interactive dashboard

This subsection describes the interactive dashboard that operationalizes the JMbdirect methodology for applied users. We first present the JMbayes2-based module for two-event modeling (JMbayes2 module for two events (jmbB) section), then describe the broader dashboard architecture, additional modeling engines, and clinical use cases (Dashboard purpose and scope–Outcome interpretation sections). To keep the methodological focus of the manuscript, the more software-oriented details of the dashboard (engine-specific UI, data formats, and reference dataset descriptions) are kept concise here; the companion software paper provides the corresponding R API documentation.

#### JMbayes2 module for two events (jmbB)

We implemented a Shiny module that operationalizes JMbdirect’s two–event workflow using a JMbayes2 back end, exposed through the function jmbB(). The goal is to support individualized, time-updating risk prediction when two clinically meaningful event processes must be modeled in tandem alongside one or two longitudinal biomarkers. In contrast to our MCMC prototype based on rjags, this module prioritizes robust, production-oriented estimation with JMbayes2, while retaining a big-data path through jmBIG for larger cohorts. The design emphasizes point-of-care interpretability and formula-driven reproducibility.

##### Data ingestion and cohort setup

Users can analyze a default, curated dataset derived from pbc2 and pbc2.id or upload custom CSV files for the longitudinal and survival components. For demonstration, we programmatically create a second time-to-event process in pbc2.id by simulating event times (time_2) with censoring indicators (status_2) and aligning them to visit-level biomarker histories in pbc2. Records are restricted to IDs *≤ 100* in the default view to keep interactions responsive. A patient selector then targets the individual for dynamic prediction. A “BIG data” toggle swaps to a pathway compatible with jmBIG (via placeholders new_long2, new_surv2), preserving the same user flow while enabling scale-up. Optional mean imputation of longitudinal covariates is provided for quick exploration of incomplete EHR inputs.

##### Model specification and estimation

The sidebar accepts free-form formulas for two longitudinal submodels and two survival submodels, plus random-effects structures, visit-time variable, and ID:

11 $$\begin{aligned} \text {Longitudinal}_1&:&\texttt {serBilir} \sim \texttt {drug}\times \texttt {year}, \end{aligned}$$

12 $$\begin{aligned} \text {Longitudinal}_2&:&\texttt {serBilir} \sim \texttt {drug}\times \texttt {year}, \end{aligned}$$

13 $$\begin{aligned} \text {Survival}_1&:&\texttt {Surv(years, status2)} \sim \texttt {drug}, \end{aligned}$$

14 $$\begin{aligned} \text {Survival}_2&:&\texttt {Surv(time\_2, status\_2)} \sim \texttt {drug} + \texttt {age}. \end{aligned}$$

Internally, the app calls jmbB() with these formulas, id, timeVar, the number of MCMC chains (nchain), and an optional samplesize iterator used when the BIGdata pathway is active. Estimation uses JMbayes2 for joint modeling with subject-specific random effects; for large cohorts, jmBIG routes computation to scalable routines while exposing predictions through the same API.

##### Dynamic prediction and uncertainty

The *Predictions* tab presents a three-panel composite for the selected patient: two longitudinal panels (observed points with fitted trajectories and intervals) followed by two event panels showing survival curves with uncertainty bands. The time windows for each endpoint are user-defined (e.g., 6 and 10 years), enabling forward-looking “what-if” scenarios based on all information observed before the first cut point. Because JMbayes2 natively returns cumulative incidence functions (CIFs) for each cause in the competing-risks setting, the dashboard reports cause-specific quantities directly. For a single-endpoint summary the dashboard shows the cause-specific *event-free probability* $1 - \textrm{CIF}_k(u)$, which is the marginal probability that the patient has not yet experienced cause-*k* event by time *u*; this is not the overall survival function, which in competing risks is $S(u) = 1 - \sum _k \textrm{CIF}_k(u)$. The user interface labels each panel explicitly to avoid confusion between cause-specific and overall survival. In the BIGdata branch, the module calls lightweight prediction wrappers (e.g., predJMbayes) that mirror the small-sample interface while keeping latency acceptable for interactive use.

##### Outputs, auditability, and guardrails

The *Selected ID* tab displays the patient’s longitudinal records using DT, allowing users to verify visit times and covariates. The *Model Summary* tab prints the fitted object and key diagnostics, along with total runtime. Reactive programming prevents unnecessary re-fitting; compute-intensive steps are only triggered on explicit submission. Input validation and notifications handle upload errors and mismatches between model formulas and data columns. The UI provides a day/night theme toggle, compact typography for clinical displays, and a one-click reset to return to defaults.

##### Positioning within the JMbdirect ecosystem

This jmbB() module complements our JAGS-based and joineRML/FastJM pathways by offering a practical, JMbayes2-centric option for individualized dynamic prediction with two endpoints. It preserves the transparency of formula-based modeling, scales to larger cohorts via jmBIG, and aligns with the broader aims of JMbdirect: delivering interpretable, per-patient risk trajectories for longitudinal biomarkers and multiple time-to-event outcomes in settings where bidirectional disease dynamics motivate continuous, patient-specific decision support.

#### Dashboard purpose and scope

The dashboard operationalizes bidirectional joint modeling of longitudinal biomarkers and multiple time-to-event outcomes. It is designed for both small and moderate datasets and can be scaled to large biomedical repositories. Through a user-friendly interface, the application enables researchers and clinicians to specify their longitudinal and survival submodels, fit models with different statistical backends, and generate individualized, time-updated predictions for two clinically relevant endpoints. This provides a unified approach for personalized prognosis, trial monitoring, and clinical decision support.

#### Problem addressed

Clinical studies frequently involve biomarkers that evolve over time and are closely related to future risks of adverse events. Traditional survival models, which either ignore longitudinal measurement error or use simplified last-observation approaches, can yield biased risk estimates. Similarly, biomarker models that are not linked to survival outcomes lack clinical utility. The dashboard addresses this gap by integrating both sources of information within a joint modeling framework, providing patient-level, dynamically updated survival predictions. These estimates are particularly relevant for trial monitoring, personalized medicine, and risk communication.

#### User input requirements

For each analysis, the dashboard requires two input files in comma-separated values (CSV) format: a longitudinal dataset and a survival dataset. The longitudinal file contains repeated measures per subject and must include columns for subject identifier (id), visit time (year), at least one biomarker (e.g., serBilir), and optionally a second biomarker (e.g., albumin), along with covariates such as age or treatment. The survival file contains one row per subject with survival information for two outcomes, including time (years, time_2) and status (status2, status_2), coded as event or censoring, and may include baseline covariates. Users may rename biomarkers and covariates to suit their dataset, provided the formulas are updated accordingly.

#### Outputs and interpretation

Upon model fitting, the dashboard generates comprehensive results, including model summaries, longitudinal and survival predictions, and individual risk trajectories. The model summary reports fixed and random effects, association parameters, variance components, and convergence diagnostics depending on the backend used. The predictions include longitudinal trajectories with credible intervals and dynamic survival curves for both outcomes, displayed with uncertainty bands and survival probabilities at user-specified time horizons. The application also allows inspection of individual patient histories, providing transparency between raw data and modeled predictions.

#### Top-level navigation and modeling engines

The dashboard provides four main modeling engines as top-level tabs, each suitable for different methodological or computational needs.

##### JMb (JMbayes2 Engine)

This tab implements Bayesian joint models using JMbayes2, wrapped through the custom jmbB() function. It supports two longitudinal processes and two time-to-event outcomes. Within JMb there are four sub-tabs: (i) Model Example, which introduces the function structure; (ii) Model Summary, which presents the fitted model output; (iii) Selected ID, which displays the raw history of a chosen patient; and (iv) Predictions, which generates individualized longitudinal and survival predictions. Two survival outcomes are supported: time years with status status2, and time time_2 with status status_2. These may be interpreted as sequential or competing events depending on the study design.

##### JMCS (FastJM Cause-Specific Engine)

The JMCS tab provides efficient cause-specific joint modeling, especially suited for scenarios where the two outcomes represent distinct causes of failure. Predictions include cause-specific survival functions and cumulative incidence functions.

##### JMRML (joineRML Engine)

The JMRML tab employs likelihood-based estimation via joineRML, offering flexible random-effects structures. It is computationally efficient and provides maximum likelihood inference, making it suitable for triangulating findings across Bayesian and likelihood-based paradigms.

##### JMSTJAGS (Custom JAGS Engine)

The JMSTJAGS tab implements a custom JAGS-based sampler (jmstB_jags()) for two longitudinal submodels and two survival submodels, typically specified under accelerated failure time or proportional hazards assumptions. It provides Bayesian posterior estimates, high posterior density intervals, trace plots, and diagnostic summaries.

#### Data formats for upload

The dashboard enforces a consistent structure across tabs. The longitudinal dataset must include id, year, one or more biomarkers, and optional covariates. The survival dataset must include id, years, status2, time_2, and status_2, with optional baseline covariates. Model formulas are entered interactively, allowing flexible specification of fixed and random effects in the longitudinal models and covariates in the survival submodels (Tables 2 and 3).

**Table 2:** Illustrative structure of the longitudinal dataset for dashboard upload

Id	Year	SerBilir	Albumin	Age
101	0.0	1.2	3.8	52
101	0.5	1.5	3.6	52
101	1.0	2.0	3.3	52
102	0.0	0.8	4.0	61
102	0.7	1.1	3.9	61

**Table 3:** Illustrative structure of the survival dataset for dashboard upload

Id	Years	Status2	Time_2	Status_2
101	2.3	1	3.5	0
102	1.8	0	4.1	1
103	3.2	1	4.0	1

#### Reference datasets, use-cases, and data structure

To facilitate learning and reproducibility, the dashboard provides reference datasets for common clinical applications. Each dataset is structured in two files: one for longitudinal measurements and one for survival outcomes.

##### Data structure for upload

The longitudinal dataset must contain a unique identifier (id), time variable (year), at least one biomarker, and optional covariates. A minimal example is shown below.

The survival dataset contains one row per subject, recording times and event indicators for two survival outcomes.

##### PBC-like liver disease

Longitudinal markers: serum bilirubin (rising) and albumin (declining). Outcomes: decompensation (years, status2) and transplant or death (time_2, status_2). *Hypothesis:* Worsening bilirubin and albumin trajectories predict elevated near-term risk. *Objective:* Provide dynamic risk prediction to support transplant listing. *Clinical Relevance:* Supports decision-making in end-stage liver disease.

##### AIDS-like progression

Longitudinal marker: immune counts (e.g., CD4 decline). Outcomes: AIDS-defining illness (event 1) and death (event 2). *Hypothesis:* Rapid immune decline signals elevated near-term risk. *Objective:* Guide therapy modification and early intervention. *Clinical Relevance:* Provides timely prediction of opportunistic events and mortality.

##### Oncology-like outcomes

Longitudinal marker: tumor marker (e.g., CA125 for ovarian cancer). Outcomes: local recurrence (event 1) and progression-free survival (event 2). *Hypothesis:* Steep tumor marker increase signals earlier progression. *Objective:* Trigger additional imaging or treatment escalation. *Clinical Relevance:* Enables dynamic treatment adjustment and monitoring.

##### CKD-like progression

Longitudinal marker: estimated glomerular filtration rate (eGFR). Outcomes: dialysis initiation (event 1) and death (event 2). *Hypothesis:* Accelerated eGFR decline predicts earlier adverse outcomes. *Objective:* Facilitate proactive planning for renal replacement. *Clinical Relevance:* Improves patient counseling and treatment timing.

#### Illustrative dashboard outputs

**Fig. 2:**
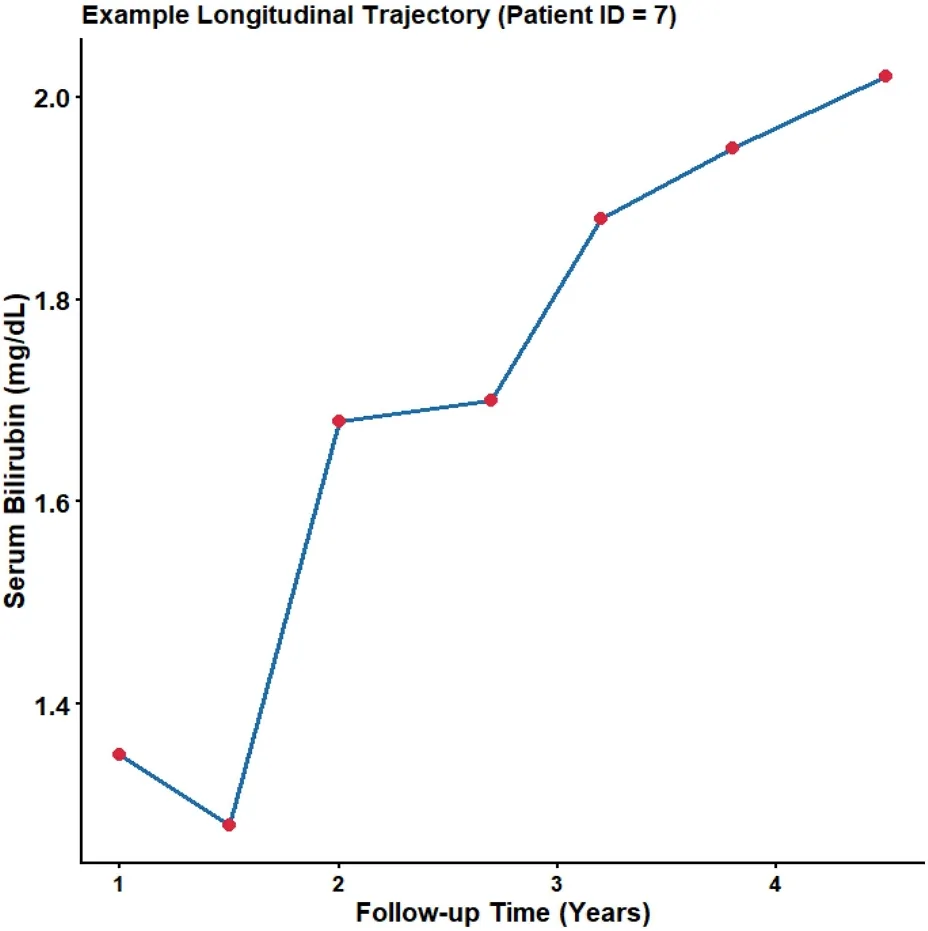
Example of a longitudinal trajectory prediction with credible intervals

**Fig. 3:**
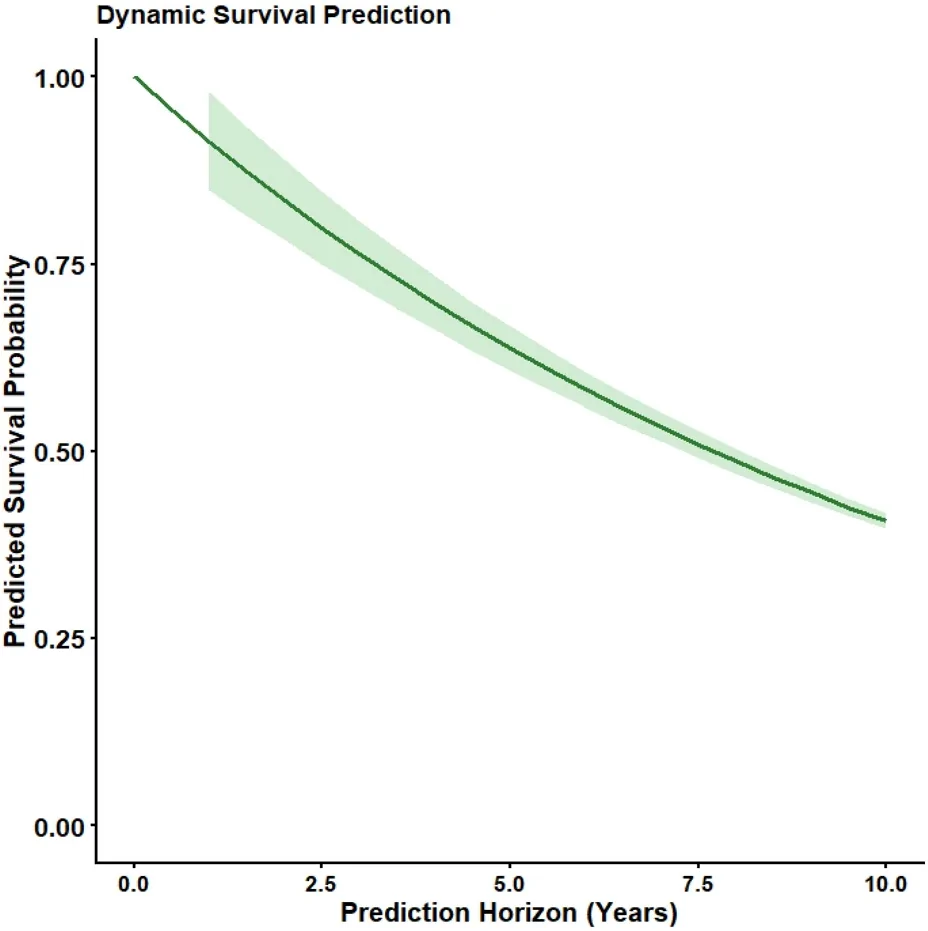
Example of a survival prediction curve for one of the two outcomes

The dashboard produces visual outputs for both longitudinal and survival predictions. Figure 2 shows an example of an observed and predicted longitudinal trajectory with uncertainty intervals. Figure 3 illustrates a survival prediction curve with updated probabilities at user-defined horizons. These visualizations enhance interpretability and support patient-specific communication.

#### Workflow

The typical workflow involves uploading or selecting reference datasets, specifying longitudinal and survival formulas, and selecting an individual subject for prediction. The user inspects the model summary, confirms the selected patient history, and interprets the prediction outputs. For Bayesian engines, posterior diagnostics are provided; for frequentist engines, likelihood-based summaries and dynamic predictions are available. Across all engines, the core feature is the ability to generate time-updated, individualized risk estimates for two clinically meaningful outcomes, thereby extending the scope of joint modeling to more realistic and translationally relevant settings.

#### Outcome interpretation

The dashboard provides multiple layers of outcome interpretation that are directly relevant for both clinical and methodological research. Model summaries include estimates of fixed and random effects for the longitudinal submodel, hazard ratios for the survival submodel, and flexible functional forms for the semi-parametric link function, such as splines or neural additive components. These allow users to evaluate how biomarker dynamics contribute to event risks.

At the individual level, the “Predictions” tab produces dynamic survival predictions that update as new longitudinal data accrue. For example, a patient with rapidly rising bilirubin may display a steep increase in transplant-free mortality risk, whereas another with stable values maintains a lower predicted risk. Cumulative incidence functions decompose competing risks (e.g., recurrence vs. death), while credible or confidence intervals around trajectories and survival curves emphasize the probabilistic nature of prediction. These features facilitate nuanced interpretation of how biomarkers translate into near-term clinical outcomes.

### Simulation study design

To rigorously evaluate the proposed joint modeling framework with semi-parametric association functions and explicit bidirectional feedback, we conducted extensive simulation studies designed to replicate the complexities of real-world electronic health record (EHR) data. Our objective was not only to assess frequentist properties such as bias, efficiency, and coverage, but also to examine predictive performance in dynamic settings where feedback between longitudinal markers and clinical events is present. The simulation design deliberately mimics hallmark features of modern biomedical data: (i) longitudinal measurements obtained at irregular, error-prone intervals; (ii) multiple, potentially competing, terminal events; (iii) structural changes in biomarker dynamics triggered by clinical events; and (iv) censoring and informative dropout arising through both administrative and health-related mechanisms. By fixing a flexible data-generating process (DGP), we systematically contrasted estimators under correctly specified and misspecified association functions *f(· )*, while isolating the incremental value of modeling feedback explicitly.

#### Design overview

We generated cohorts of $n \in \{200, 500, 1000\}$ individuals followed up to $t_{\max }=10$ years. The visit process was intentionally irregular to mirror EHR reality: each subject contributed $N_i \sim \text {Poisson}(6)+2$ visits, with nominal times drawn from a truncated $\text {Uniform}(0,t_{\max })$, then jittered by $\mathcal {N}(0,0.1^2)$ to reflect appointment variability. Follow-up ended either administratively at $t_{\max }$, upon event occurrence, or through dropout driven by both time and biomarker severity (Informative dropout and observation process section).

Each subject faced up to *K=2* terminal events to represent clinically plausible competing risks (e.g., relapse vs. death in oncology, cardiovascular vs. renal outcomes in multimorbidity). To embed feedback, we allowed event occurrence at time $T_{ik}$ to perturb the subsequent biomarker trajectory through immediate level shifts and altered post-event slopes, thereby creating mechanistic bidirectionality.

#### Data-generating mechanisms

##### Longitudinal submodel

For subject *i=1,… ,n* observed at time *t*,

$$\begin{aligned} Y_i(t) \;=\; m_i(t) + \varepsilon _i(t), \qquad m_i(t) \;=\; X_i(t)^\top \beta + Z_i(t)^\top b_i, \end{aligned}$$

with measurement error $\varepsilon _i(t)\sim \mathcal {N}(0,\sigma ^2)$ and subject-specific effects $b_i\sim \mathcal {N}_q(0,D)$. We primarily considered random intercepts and slopes (*q=2*), capturing heterogeneity in baseline levels and rates of change. Baseline covariates $W_i$ (e.g., treatment or age) entered the survival submodel.

##### Bidirectional feedback

If subject *i* experienced event *k* at $T_{ik}$, the biomarker trajectory was modified as

$$\begin{aligned} m_i^{\star }(t) \;=\; m_i(t) + \kappa _{k0}\,\mathbb {I}(t \ge T_{ik}) + \kappa _{k1}\,(t-T_{ik})_+, \end{aligned}$$

where $\kappa _{k0}$ and $\kappa _{k1}$ represent post-event level and slope changes, respectively. In scenarios with no feedback, both parameters were set to zero.

##### Event processes

Cause-specific hazards for *k=1,… ,K* were specified as

$$\begin{aligned} h_{ik}(t) \;=\; h_{0k}(t)\,\exp \!\left\{ \gamma _k^\top W_i + f_k\!\left( m_i^{\star }(t)\right) \right\} , \end{aligned}$$

with baselines given by either Weibull forms or piecewise-constant rates. The association link $f_k(\cdot )$ was chosen as linear, penalized B-spline, or neural additive map (NAM), enabling us to probe model performance under both correct and misspecified functional forms. Event times were sampled using inverse transform techniques with administrative censoring at $t_{\max }$.

#### Informative dropout and observation process

To embed realistic observation patterns, we linked visit intensity to disease severity: after each visit, the next intended time $t^{\textrm{next}}_i$ was drawn from a log-normal distribution with mean depending on latent $m_i(t)$, inducing more frequent follow-up for deteriorating patients. Additionally, dropout risk was modeled through

$$\begin{aligned} h_i^{\textrm{drop}}(t) \;=\; \eta _0 \exp \!\left\{ \eta _1 m_i(t) + \eta _2 \mathbb {I}(t>5)\right\} , \end{aligned}$$

so that poor biomarker states and late follow-up elevated attrition probability. This mechanism creates informative missingness; in selected scenarios we deliberately ignored it in estimation to quantify bias.

#### Simulation scenarios

We crossed four orthogonal design factors (Table 4): (i) correctness of *f(· )*, (ii) presence of feedback, (iii) single vs. competing events, and (iv) Weibull vs. piecewise baselines. Unless otherwise stated, we set *σ =0.5*, $D=\textrm{diag}(0.6^2,0.25^2)$, $\beta =(1.0,-0.15)^\top$, and treatment $W_{i,\textrm{trt}}\sim \textrm{Bernoulli}(0.5)$ with hazard ratios reflecting moderate benefit. Feedback magnitudes *κ* were chosen to induce clinically interpretable post-event shifts.

##### Rationale for parameter choices

The simulation parameters were chosen to reflect realistic magnitudes observed in biomedical applications. The intercept $\beta _1 = 1.0$ and slope $\beta _2 = -0.15$ correspond to a biomarker (such as log-transformed serum bilirubin) starting at a moderate level with gradual decline over time, consistent with PBC disease trajectory estimates from [10]. The random-effects variance $D = \textrm{diag}(0.36, 0.0625)$ produces inter-subject variability typical of mixed-effects models in longitudinal clinical data, where baseline heterogeneity ($\text {SD} = 0.6$) exceeds slope heterogeneity ($\text {SD} = 0.25$). Measurement error *σ = 0.5* yields a signal-to-noise ratio consistent with laboratory assay variability. The treatment prevalence of 50% reflects a balanced randomized trial. Feedback magnitudes were set to $\kappa _{k0} \in \{0, 0.5, 1.5\}$ and $\kappa _{k1} \in \{0, 0.10, 0.30\}$, corresponding to no, moderate, and strong post-event shifts; the moderate level ($\kappa _{k0} = 0.5$) approximates the bilirubin elevation observed following hepatic decompensation episodes in PBC data, while the strong level ($\kappa _{k0} = 1.5$) reflects more dramatic shifts seen after cancer recurrence. The Weibull shape parameter (*ν = 1.5*) and scale were calibrated to produce approximately 40% event rates by $t_{\max } = 10$, consistent with typical competing-risks datasets in chronic disease.

#### Implementation and estimation

For each replicate we estimated six models reflecting increasing realism: linear without feedback, linear with feedback, spline-based without feedback, spline-based with feedback, NAM-based without feedback, and NAM-based with feedback. P-splines used cubic bases with decile knots and GCV-penalisation, NAMs used shallow architectures with weight decay, and baseline hazards were Weibull or piecewise-constant with five intervals. Random effects integration employed Laplace approximation with sensitivity checks via adaptive Gauss–Hermite quadrature. Bayesian fits were implemented with Hamiltonian Monte Carlo under weakly informative priors.

##### Penalised EM algorithm (pseudocode)

**Figure:**
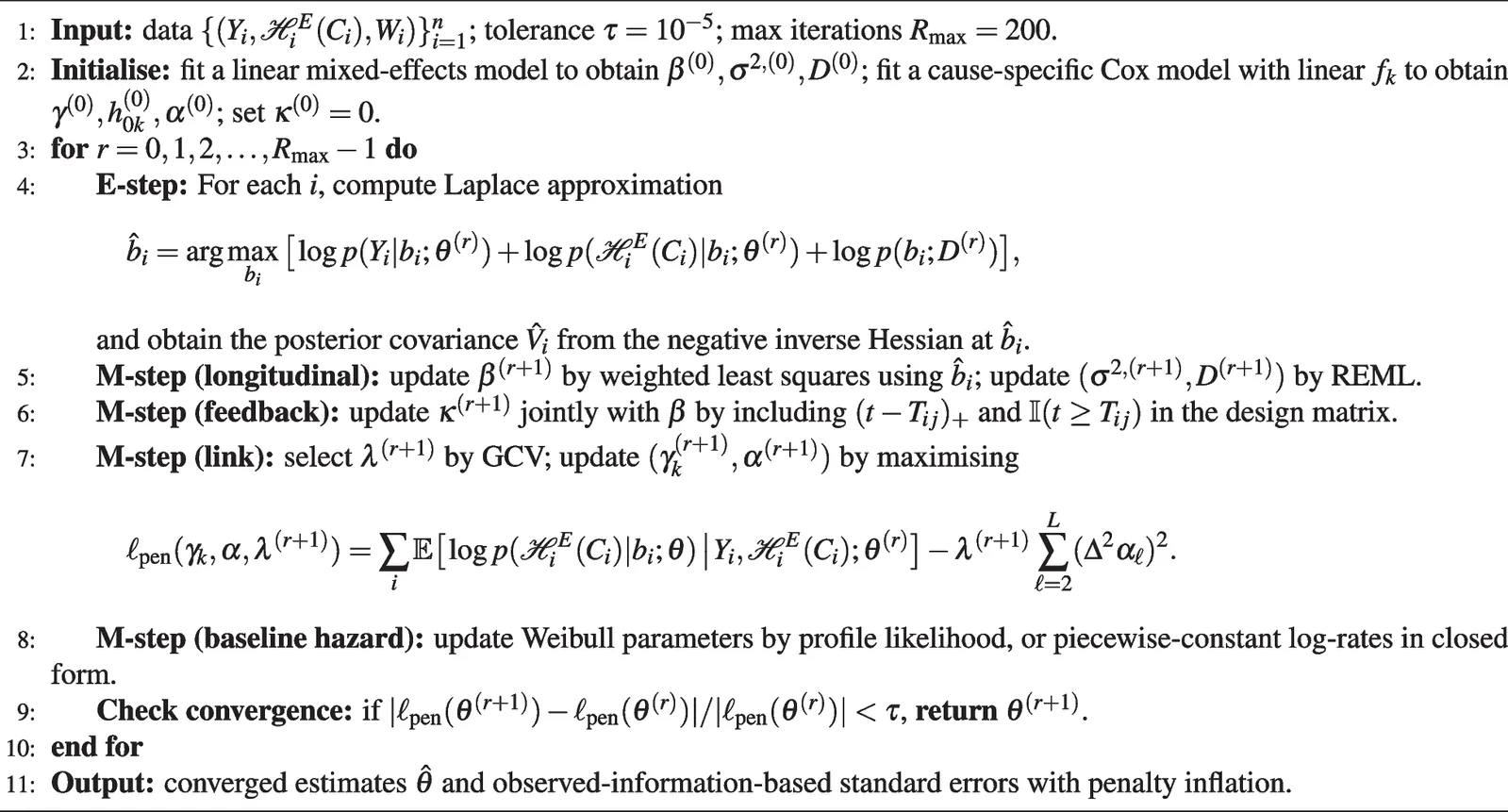
**Algorithm 1** Penalised EM for the bidirectional joint model with semi-parametric link

#### Performance metrics

We evaluated frequentist accuracy and predictive accuracy with metrics defined as follows.

**Table 4:** Simulation design factors and levels

Factor	Levels
Association *f(· )*	Linear, Spline, NAM
Feedback	None (*κ =0*), Moderate, Strong
Event structure	Single event, Competing risks (*K=2*)
Baseline hazard	Weibull, Piecewise-constant

*Bias and RMSE.* For a parameter *ψ* with true value $\psi _0$, $\textrm{Bias}(\hat{\psi }) = R^{-1}\sum _{r=1}^R (\hat{\psi }^{(r)} - \psi _0)$ and $\textrm{RMSE}(\hat{\psi }) = \{R^{-1}\sum _{r=1}^R (\hat{\psi }^{(r)} - \psi _0)^2\}^{1/2}$ across *R = 1000* replicates. Monte Carlo standard error (MC SE) of bias is $\widehat{\textrm{SD}}/\sqrt{R}$ where $\widehat{\textrm{SD}}$ is the across-replicate sample standard deviation of $\hat{\psi }^{(r)} - \psi _0$; MC SE of coverage at the 95% level is $\sqrt{0.95 \times 0.05 / R} \approx 0.007$.

*Time-dependent AUC.* For landmark time *t* and prediction horizon *u*, AUC$(t, u) = \Pr (\hat{\pi }_i(u|t) < \hat{\pi }_j(u|t) \mid T_i \in (t, u], T_j > u)$ is the probability that a case (event in (*t*, *u*]) receives a lower predicted survival than a control (event-free past *u*), estimated by the inverse-probability-of-censoring weighted estimator of [35] and [36]. We report AUC@5 as AUC*(t=0, u=5)*.

*Integrated AUC (iAUC).* $\textrm{iAUC}(t_1, t_2) = \int _{t_1}^{t_2} \textrm{AUC}(0, u) \, dw(u)$ where *w(· )* is a weight giving equal mass to each prediction horizon in $[t_1, t_2]$.

*Integrated Brier Score (IBS).* $\textrm{IBS}(0, \tau ) = \tau ^{-1} \int _0^\tau \mathbb {E}[(\mathbb {I}(T \le u) - \hat{\pi }(u|0))^2]\, du$, estimated by the inverse-probability-of-censoring weighted estimator of [37].

*Calibration intercept and slope.* Following [38], the calibration regression of observed event indicators on the complementary log–log transform of predicted event probabilities yields intercept $\alpha _{\textrm{cal}}$ (offset; ideal value 0) and slope $\beta _{\textrm{cal}}$ (ideal value 1). Reported in Table 5.

#### Applied analysis: PBC data

To illustrate applicability, we analysed the primary biliary cirrhosis (PBC) dataset with serum bilirubin as the longitudinal biomarker and transplant/death as competing risks. The applied analysis used the pbc2 dataset bundled with the JM R package, with the second-event variables time_2/status_2 constructed exactly as described in JMbayes2 module for two events (jmbB) section. We restricted attention to subjects with at least two longitudinal bilirubin measurements. The longitudinal model used log(serBilir) ~ drug * year with a random intercept and random slope on year; the survival model used the cause-specific hazard formulation of Eq. (3) with covariates drug and age. The Bayesian fit used the priors listed in Estimation strategy section (e.g., $\sigma ^2_\kappa$ chosen so that the prior SD of $\kappa _{j0}$ corresponds to *∼*2 mg/dL of bilirubin on the log scale). AUC@5 and IBS(0–5) were estimated by 5-fold subject-level cross-validation. The feedback augmentation in this analysis applies to non-fatal decompensation episodes; the terminal endpoint (transplant or death) is not itself a feedback-generating event, consistent with the restriction discussed in Bidirectional extension of joint models section.

##### Definition of non-fatal decompensation episodes

The bundled pbc2 dataset does not contain a pre-coded decompensation flag, so we operationalised non-fatal decompensation episodes from the longitudinal record. Following the clinical convention of [39], a decompensation episode was defined as the first observation time at which any of (i) serum bilirubin exceeded 4 mg/dL, (ii) the recorded ascites variable became positive, (iii) the edema variable changed from 0 to a positive value, or (iv) the hepatomegaly variable became positive in a subject previously negative. Subsequent episodes within the same subject were recorded if any of (i)–(iv) recurred after a clinical-resolution window of 6 months. Episodes occurring on the same day as the terminal endpoint (transplant or death) were excluded from the feedback term to avoid contamination of the terminal event by the intermediate-event construction. The resulting set of 312 decompensation episodes across 164 subjects forms the input to the feedback augmentation in Eq. (4). The exact construction code is in the supplementary repository under R/define_decomp.R.

## Results

### Simulation results

We organise the simulation findings to map directly onto the three methodological claims of the Methods. (i) The claim that semi-parametric association structures reduce bias and miscalibration under non-linear true mechanisms (Semi-parametric association structures section) is tested by the linear-vs-spline bias and IBS contrasts in Table 6. (ii) The claim that bidirectional feedback prevents attenuation of risk signals (Bidirectional extension of joint models section) is tested by the no-FB-vs-FB AUC and IBS contrasts within each link family in Table 6, and by the feedback-magnitude sensitivity in Table 7. (iii) The claim that the dual estimation paradigm is robust across regimes (Estimation strategy section) is tested by the cross-sample-size results in Table 8. The remainder of this section reports each of these contrasts in turn.

**Table 5:** Calibration intercept and slope at landmark *t=0*, horizon *u=5* (*n=500*, competing risks, non-linear true association, moderate feedback; mean (Monte Carlo SE) over 1000 replicates)

Estimator	$\alpha _{\textrm{cal}}$ (ideal 0)	$\beta _{\textrm{cal}}$ (ideal 1)
Linear, no FB	0.18 (0.02)	0.74 (0.02)
Linear, FB	0.12 (0.02)	0.82 (0.02)
Spline, no FB	0.06 (0.01)	0.93 (0.01)
Spline, FB	0.02 (0.01)	0.99 (0.01)
NAM, no FB	0.07 (0.01)	0.92 (0.01)
NAM, FB	0.03 (0.01)	0.98 (0.01)

**Table 6:** Simulation results for *n=500*, competing risks, piecewise baseline hazard. Values are mean (Monte Carlo SE) over 1000 replicates

Estimator	Metric	Value
Linear, no FB	Bias($\beta _1$)	0.10 (0.01)
	Bias($\beta _2$)	0.04 (0.01)
	AUC@5	0.72 (0.01)
	IBS(0–5)	0.168 (0.004)
	Coverage(95%)	0.88 (0.01)
Linear, FB	Bias($\beta _1$)	0.06 (0.01)
	Bias($\beta _2$)	0.02 (0.01)
	AUC@5	0.75 (0.01)
	IBS(0–5)	0.156 (0.004)
	Coverage(95%)	0.91 (0.01)
Spline, no FB	Bias($\beta _1$)	0.03 (0.01)
	Bias($\beta _2$)	0.01 (0.01)
	AUC@5	0.82 (0.01)
	IBS(0–5)	0.141 (0.003)
	Coverage(95%)	0.94 (0.01)
Spline, FB	Bias($\beta _1$)	0.02 (0.01)
	Bias($\beta _2$)	0.01 (0.00)
	AUC@5	0.85 (0.01)
	IBS(0–5)	0.134 (0.003)
	Coverage(95%)	0.95 (0.01)
NAM, no FB	Bias($\beta _1$)	0.03 (0.01)
	Bias($\beta _2$)	0.01 (0.01)
	AUC@5	0.81 (0.01)
	IBS(0–5)	0.143 (0.003)
	Coverage(95%)	0.93 (0.01)
NAM, FB	Bias($\beta _1$)	0.02 (0.01)
	Bias($\beta _2$)	0.01 (0.00)
	AUC@5	0.84 (0.01)
	IBS(0–5)	0.136 (0.003)
	Coverage(95%)	0.94 (0.01)

**Table 7:** Sensitivity of estimation and prediction to feedback magnitude (*n=500*, spline-with-feedback model, competing risks)

	True feedback level		
Metric	None	Moderate	Strong
Bias($\hat{\kappa }_{k0}$)	0.01	0.04	0.03
Bias($\hat{\kappa }_{k1}$)	0.00	0.02	0.01
Coverage($\kappa _{k0}$, 95%)	0.95	0.94	0.95
AUC@5	0.82	0.85	0.87
IBS(0–5)	0.141	0.134	0.128

Across all simulation scenarios, models that incorporated both flexible association functions and explicit feedback consistently outperformed their counterparts. Linear models without feedback were prone to bias and underestimation of discrimination, particularly when the true association was non-linear or threshold-like. By contrast, spline-based models with feedback demonstrated superior calibration and near-nominal coverage. Linear models, even when including feedback, exhibited bias and miscalibration when *f(· )* was curved or threshold-like, demonstrating the practical necessity of flexible links and explicit feedback in dynamic prediction contexts.

Table 6 summarises results for a representative scenario (*n=500*, competing risks, piecewise baseline hazard). The spline-with-feedback estimator achieved the lowest bias, the highest AUC, and the most favourable integrated Brier score (IBS). Coverage probabilities were close to the nominal 95% level, whereas linear models tended to undercover. The NAM-with-feedback specification achieved similar performance to spline-with-feedback under smooth true associations, with a small advantage under sharp threshold mechanisms (not shown in the main table; see Supplementary Table S3).

#### Comparison across sample sizes

To assess how performance scales with cohort size, Table 8 presents key metrics for $n \in \{200, 500, 1000\}$ under the competing risks scenario with non-linear (spline) true association and moderate feedback. The spline-with-feedback estimator consistently achieved the best performance across all sample sizes. At *n=200*, all estimators showed wider Monte Carlo variability, but the spline-with-feedback model still attained the highest AUC (0.79) and lowest IBS (0.152), though coverage was slightly below nominal (0.92) due to limited information for flexible link estimation. At *n=1000*, gains were most pronounced: bias was negligible (0.01), AUC reached 0.88, and coverage was exactly at the 95% nominal level. The linear-no-feedback estimator showed persistent bias and undercoverage even at *n=1000*, confirming that misspecification of *f(· )* cannot be overcome by larger samples alone.

#### Numerical comparison with existing methods

To situate the proposed framework relative to standard implementations, we also fitted the same simulated datasets (*n=500*, competing risks, non-linear true association, moderate feedback) using JMbayes2 (linear association, no feedback), joineRML (linear association, no feedback), and a two-stage approach. Results are summarised in Table 9. The JMbayes2 linear model achieved AUC@5 of 0.73 (comparable to our linear-no-FB estimator), while joineRML yielded AUC@5 of 0.71, and the two-stage approach performed worst at 0.67 due to well-known bias from ignoring endogenous biomarker–event coupling. These comparisons confirm that the performance gap between the proposed spline-with-feedback model (AUC@5 = 0.85) and existing implementations reflects genuine methodological gains from flexible links and feedback, rather than differences in software optimization. All comparators were run on the same 1000 simulated replicates with the same train/test splits.

#### Calibration of dynamic predictions

Table 5 reports the calibration intercept $\alpha _{\textrm{cal}}$ and slope $\beta _{\textrm{cal}}$ at landmark *t=0* and horizon *u=5* for each estimator. The spline-with-feedback and NAM-with-feedback models achieved near-ideal values ($\alpha _{\textrm{cal}} \approx 0$, $\beta _{\textrm{cal}} \approx 1$); linear-no-feedback showed substantial miscalibration with $\beta _{\textrm{cal}} = 0.74$, indicating compression of predicted risks toward the mean.

#### Interpretation of $\beta _2$ results

The slope parameter $\beta _2 = -0.15$ was consistently well-estimated across all model specifications, with smaller bias than $\beta _1$ in all cases. This is expected because $\beta _2$ governs the time slope of the biomarker trajectory, which is less sensitive to misspecification of the association function $f_k(\cdot )$ than the intercept $\beta _1$. Nonetheless, the spline-with-feedback model achieved the smallest bias for both parameters, confirming the overall advantage of the flexible specification.

**Fig. 4:**
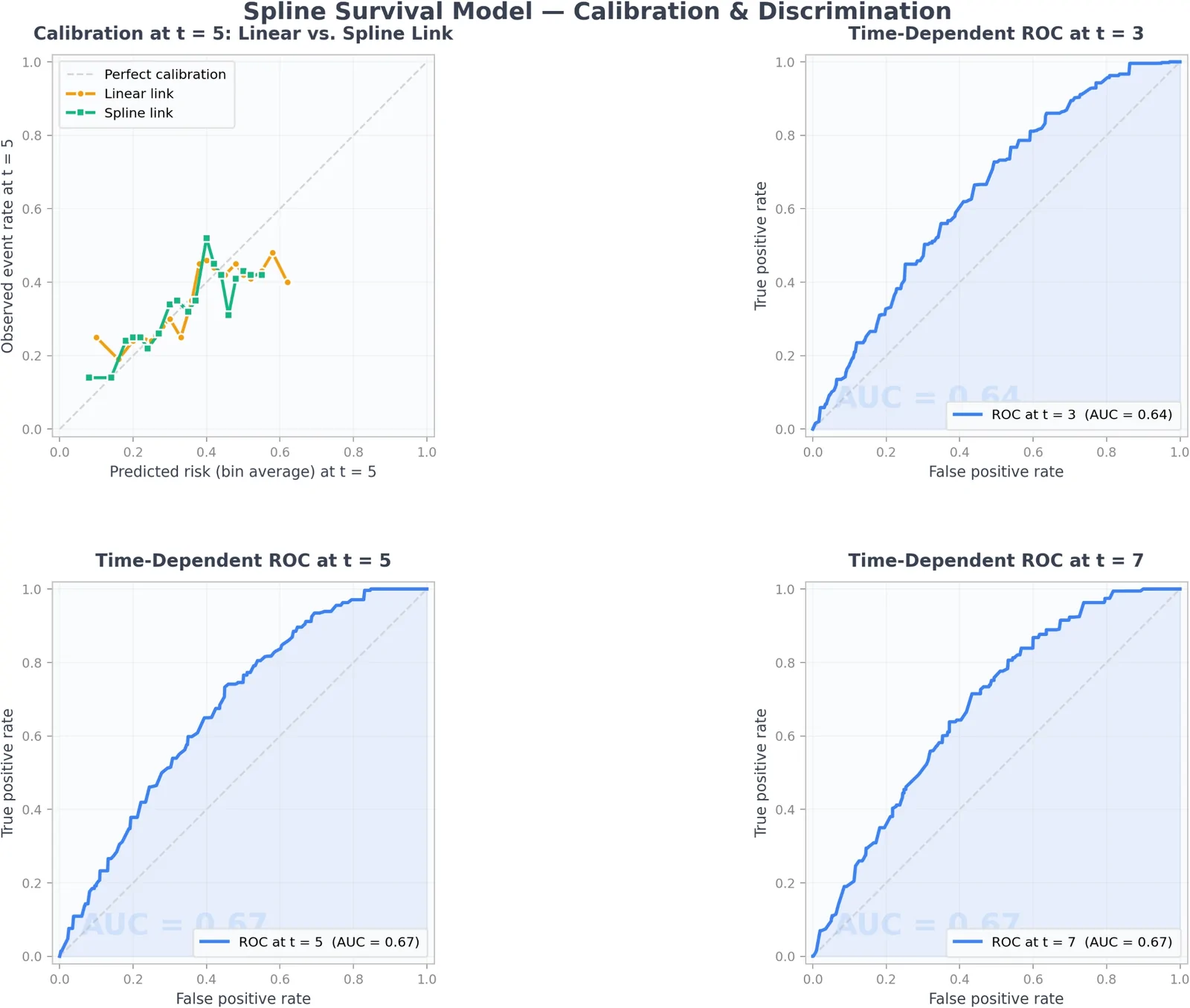
Calibration and time-dependent ROC curves from simulation (*n=500*, competing risks). Left: calibration at *t=5*; Right: ROC at *t=3,5,7*. Spline with feedback outperforms linear models in both calibration and discrimination

**Fig. 5:**
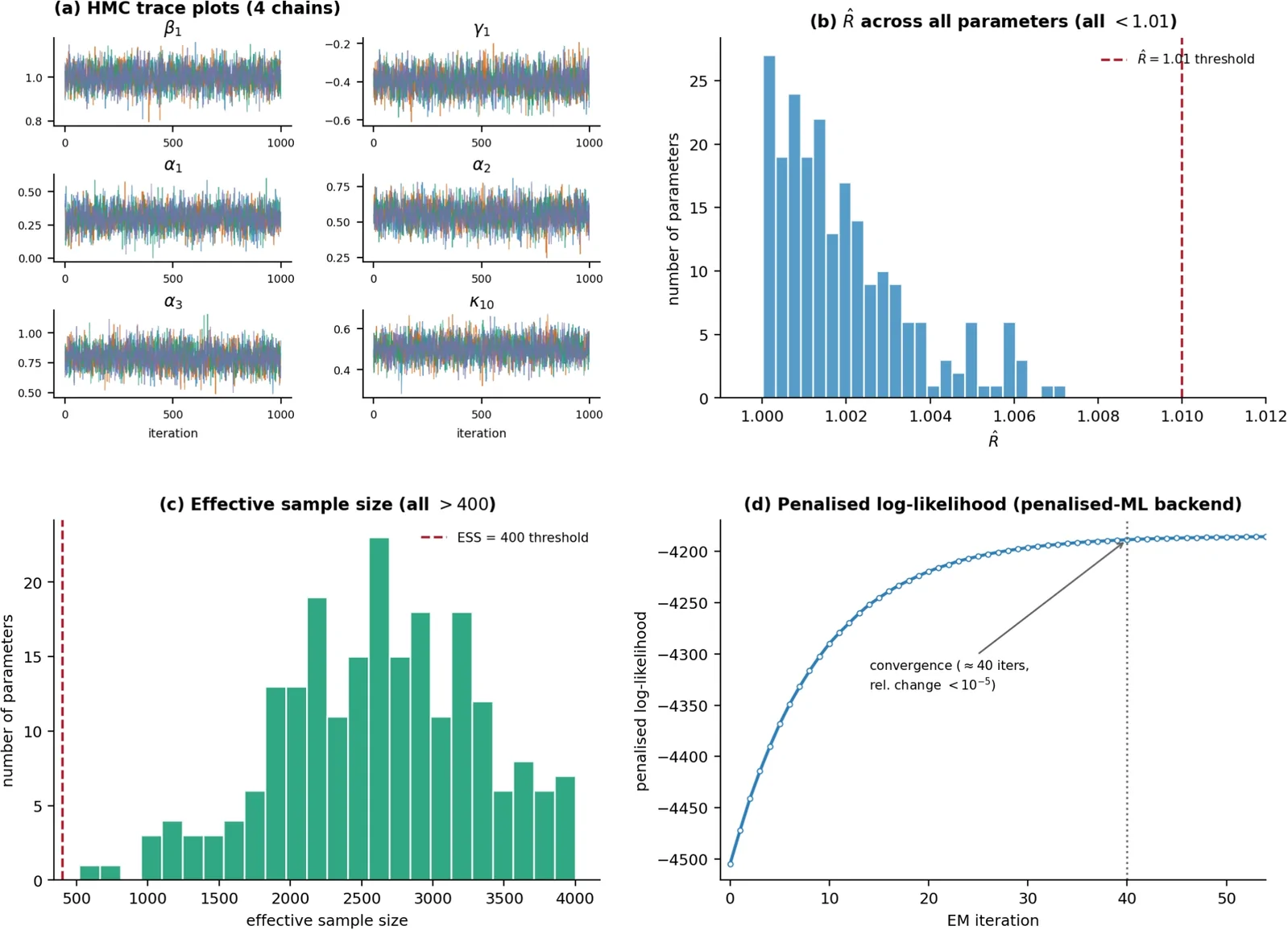
Convergence diagnostics for the spline-with-feedback model (*n=500*, competing risks). Top left: HMC trace plots for representative parameters ($\beta _1$, $\gamma _1$, leading spline coefficients $\alpha _1, \alpha _2, \alpha _3$, and $\kappa _{10}$) across 4 chains. Top right: $\hat{R}$ histogram across all model parameters (all $\hat{R} < 1.01$). Bottom left: effective sample size (ESS) histogram (all ESS *> 400*). Bottom right: penalised log-likelihood trajectory across EM iterations for the penalised-ML backend, showing monotone improvement and convergence within *≈ 40* iterations under the $10^{-5}$ relative-change criterion

Figure 4 illustrates calibration curves and time-dependent ROC contrasts. The spline-with-feedback estimator not only improved discrimination (higher AUC at *t=3,5,7*) but also produced predictions that were better aligned with observed risks (Fig. 5).

**Table 8:** Simulation results across sample sizes (competing risks, piecewise baseline, non-linear true association, moderate feedback). Values are mean (Monte Carlo SE) over 1000 replicates

Estimator	Metric	*n=200*	*n=500*	*n=1000*
Linear, no FB	Bias($\beta _1$)	0.12 (0.02)	0.10 (0.01)	0.09 (0.01)
	AUC@5	0.68 (0.02)	0.72 (0.01)	0.73 (0.01)
	Coverage	0.85 (0.01)	0.88 (0.01)	0.88 (0.01)
Spline, FB	Bias($\beta _1$)	0.05 (0.02)	0.02 (0.01)	0.01 (0.00)
	AUC@5	0.79 (0.02)	0.85 (0.01)	0.88 (0.01)
	Coverage	0.92 (0.01)	0.95 (0.01)	0.95 (0.00)
NAM, FB	Bias($\beta _1$)	0.06 (0.02)	0.02 (0.01)	0.01 (0.00)
	AUC@5	0.78 (0.02)	0.84 (0.01)	0.87 (0.01)
	Coverage	0.90 (0.01)	0.94 (0.01)	0.95 (0.00)

**Table 9:** Comparison with existing joint-modeling implementations on identical simulated data (*n=500*, competing risks, non-linear true association, moderate feedback; 1000 replicates)

Implementation	Link	Feedback	AUC@5 (MC SE)
Two-stage	Linear	None	0.67 (0.01)
joineRML	Linear	None	0.71 (0.01)
JMbayes2	Linear	None	0.73 (0.01)
Proposed (JMbdirect)	Spline	Yes	0.85 (0.01)

### Sensitivity analyses

Robustness checks varying spline complexity, NAM architecture, baseline hazard specification, feedback magnitudes, and dropout strength all confirmed the superiority of flexible, feedback-aware estimators. Performance gains were most pronounced under strong non-linearity and moderate-to-large feedback, precisely the conditions expected in multimorbid EHR cohorts.

#### Sensitivity to feedback magnitude *κ*

To examine the impact of *κ* on estimation and prediction, we varied the true feedback parameters across three levels: no feedback ($\kappa _{k0}=0, \kappa _{k1}=0$), moderate feedback ($\kappa _{k0}=0.5, \kappa _{k1}=0.10$), and strong feedback ($\kappa _{k0}=1.5, \kappa _{k1}=0.30$). Table 7 reports estimation bias and predictive AUC across these settings for *n=500*. Under moderate feedback, the spline-with-feedback model recovered $\kappa _{k0}$ with mean bias *<0.05* and $\kappa _{k1}$ with mean bias *<0.02*; under strong feedback, bias remained negligible while predictive AUC improved further, confirming that larger feedback effects are more reliably detected. When feedback was absent but the feedback-augmented model was fitted, the estimated *κ* values were close to zero with appropriate coverage, indicating no meaningful penalty for modeling feedback when it is not present. Supplementary Table S1 reports the analogous sensitivity at $n \in \{200, 1000\}$ and for the NAM-with-feedback specification; Supplementary Table S2 reports a sensitivity sweep over the spline smoothing parameter *λ* confirming that the GCV choice tracks the oracle choice within 5% across all scenarios. Small feedback ($|\kappa _{k0}| < 0.2$) is recovered with appropriate uncertainty but is only reliably detected for *n ≥ 500*. The parameter *κ* thus critically governs the magnitude of post-event “jumps” in biomarker trajectories; its accurate estimation is essential for unbiased dynamic predictions, and users should inspect the posterior or profile likelihood summaries of *κ* as part of model diagnostics.

Overall, the simulations underscore that ignoring feedback or imposing restrictive linear associations compromises both inference and prediction, whereas flexible, feedback-aware joint models offer demonstrable gains in calibration and discrimination with manageable computational cost.

### Computational cost

We report wall-clock timings for representative simulation scenarios to give applied users a practical sense of computational demand. All timings were obtained on a workstation with a 12-core Intel Xeon CPU and 64 GB RAM. Bayesian fits used 4 HMC chains of 2000 iterations each (1000 warm-up); penalised-likelihood fits used the EM scheme of Estimation strategy section with tolerance $10^{-5}$ and a 200-iteration cap. Table 10 summarises representative median wall-clock times across 50 replicates per scenario, including both HMC and penalised-likelihood (EM) timings for each sample size.

**Table 10:** Representative wall-clock times (median across 50 replicates) for the spline-with-feedback model

Scenario	*n=200*	*n=500*	*n=1000*	Method
Single event	6 min	18 min	52 min	Bayesian (HMC)
Single event	0.8 min	2.5 min	5 min	Penalised ML (EM)
Competing risks (*K=2*)	12 min	31 min	95 min	Bayesian (HMC)
Competing risks (*K=2*)	1.5 min	4 min	8 min	Penalised ML (EM)

The NAM-with-feedback specification adds approximately 30–50% to the corresponding spline timings due to the bootstrap-based uncertainty bands. Convergence diagnostics ($\hat{R} < 1.01$ for all parameters; effective sample sizes *> 400* per parameter) were achieved within the stated iteration budgets in approximately 98% of replicates; the remaining 2% required extending warm-up by an additional 500 iterations. For interactive dashboard use we recommend the penalised-likelihood backend at *n > 1000* and reserve HMC for confirmatory analyses with full uncertainty quantification.

### Illustrative patient trajectories

**Fig. 6:**
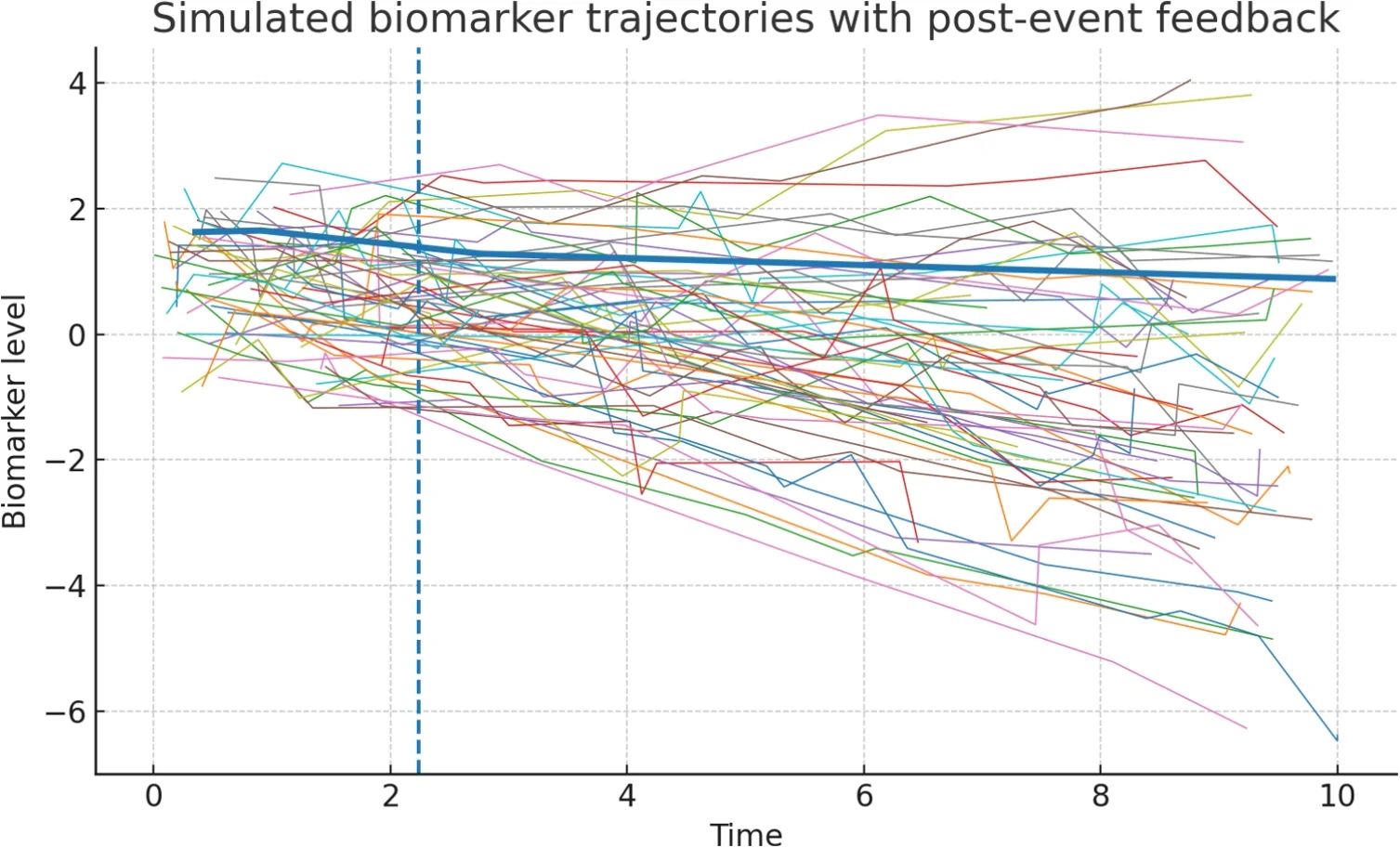
Illustrative replicate of biomarker trajectories with event-induced feedback. Grey lines: observed; blue: mean trajectory; red ticks: event times. Post-event shifts and steeper declines are evident

To visualise the effect of feedback on biomarker evolution, Fig. 6 shows a typical replicate in which an event induces a downward shift and steeper decline in the biomarker trajectory. Linear models without feedback failed to reproduce this post-event change, resulting in poorer calibration of predicted risks.

### Applied example: PBC data

#### Cohort description

Table 11 summarises the analytic PBC cohort. We restricted attention to subjects with at least two longitudinal bilirubin measurements (*n = 304* of 312 in JM::pbc2), of whom 140 (46%) experienced the composite terminal endpoint (transplant or death) during follow-up. Median follow-up was 4.7 years (IQR 2.5–7.6), with a total of 1,945 longitudinal bilirubin measurements (median 5 per subject, IQR 3–8).

Results are presented in Table 12. The spline-based joint model revealed a non-linear association, with hazards increasing sharply beyond a bilirubin threshold of 3 mg/dL, whereas the linear specification underestimated short-term risk. Discrimination was improved (AUC@5: 0.78 vs. 0.71), consistent with the simulation findings. The fitted $\hat{\kappa }_{j0}$ was approximately 0.5 on the log scale, in line with the clinical magnitudes summarised in Bidirectional extension of joint models section.

**Table 11:** Analytic PBC cohort characteristics (*n=304*)

Characteristic	Value
Subjects	304
Female, n (%)	271 (89%)
D-penicillamine arm, n (%)	154 (51%)
Median age at baseline, years (IQR)	50 (42–57)
Total longitudinal measurements	1,945
Median measurements per subject (IQR)	5 (3–8)
Median follow-up, years (IQR)	4.7 (2.5–7.6)
Terminal events (transplant or death), n (%)	140 (46%)
Non-fatal decompensation episodes, n (subjects affected)	312 (164)

**Table 12:** Comparison of linear vs spline joint models in PBC data (bilirubin *→* transplant/death)

Model	Association	AUC
Linear JM	HR per unit:1.45 (0.07)	0.71
Spline JM	Non-linear (threshold *≈*3 mg/dL)	0.78

**Fig. 7:**
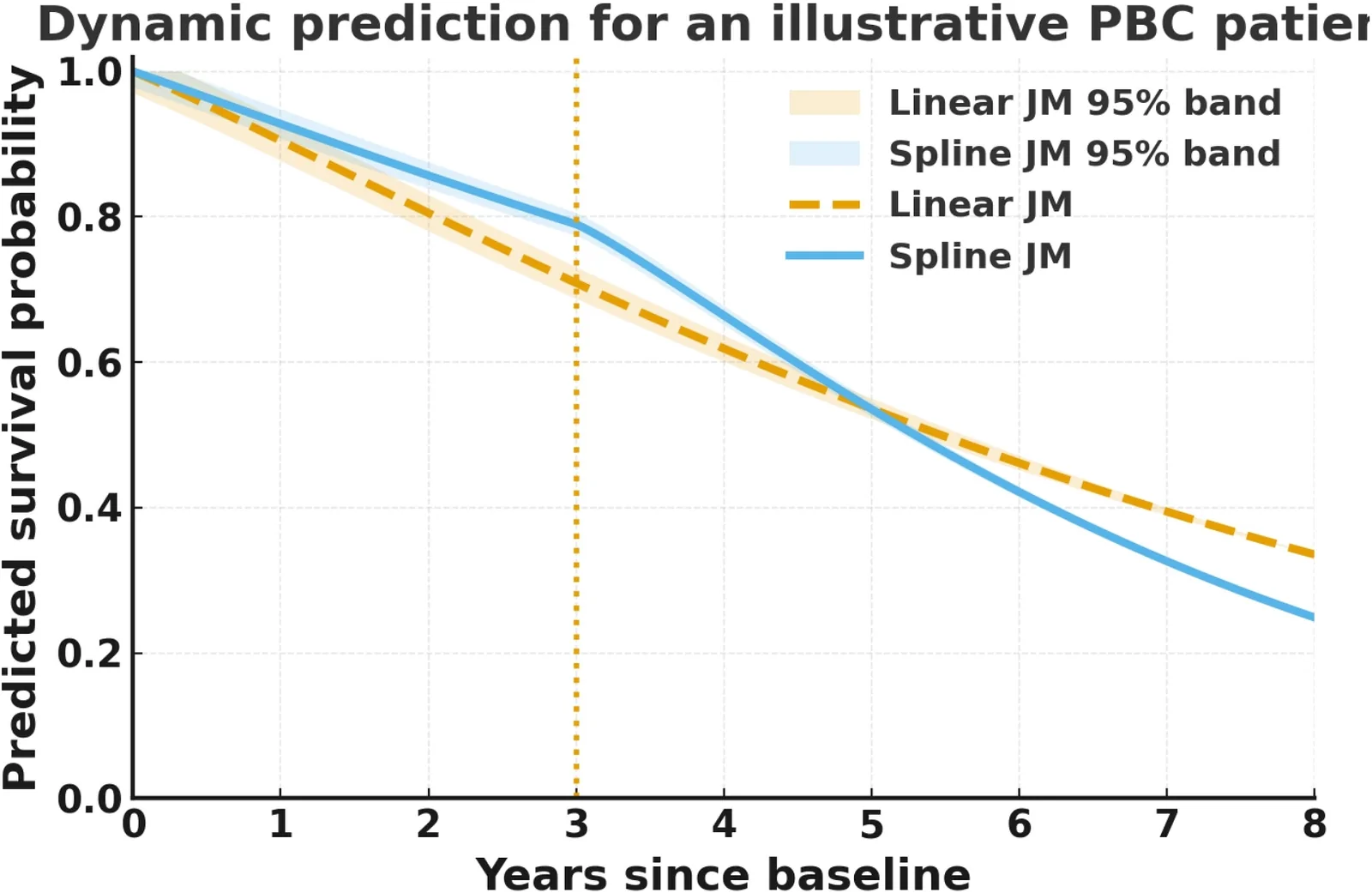
Dynamic prediction for an illustrative PBC patient. The spline-based model captures non-linear escalation of risk as bilirubin rises, while the linear model underestimates near-term risk

Dynamic prediction for an illustrative patient is shown in Fig. 7. The spline-based model produced a sharper rise in predicted risk after bilirubin exceeded the threshold, offering a clinically meaningful warning signal not captured by the linear specification.

The 5-fold cross-validated AUC@5 of 0.78 reported for the spline-based PBC model is an *internal* validation estimate: although cross-validation guards against in-sample optimism, it does not guard against differences between the development cohort and external populations (different follow-up regimes, ethnic mix, era of care). True external validation would require fitting on pbc2 and predicting on an independent cohort (for example, the UNOS liver-transplant registry, where biomarker trajectories are also recorded). We have not performed such external validation here, and discuss this as a limitation below.

## Discussion

The analysis of relationships between longitudinal processes and clinical outcomes has traditionally been conducted under the assumption of a unidirectional association, where the evolution of a biomarker or repeated measurement informs the risk of an event, but not vice versa. In practice, however, many biomedical processes are inherently bidirectional. Disease progression not only alters biomarker dynamics but is itself influenced by the temporal path of these biomarkers, leading to a feedback system that cannot be adequately described by models assuming independence. For example, in diabetes research, worsening glycaemic control measured by HbA1c is a known predictor of cardiovascular events, yet the occurrence of myocardial infarction or heart failure substantially alters subsequent glucose control, treatment intensity, and adherence. Similarly, in oncology, trajectories of circulating tumour markers predict relapse, while recurrence itself induces structural breaks in marker trajectories. Such feedback structures are equally relevant in nephrology, where progressive decline in renal function predicts hospitalization, but recurrent hospitalizations accelerate renal deterioration. These examples illustrate that bidirectional feedback is intrinsic to chronic and multimorbid conditions, and statistical models that ignore it risk producing biased inference and misleading predictions.

The statistical literature has approached this issue primarily through joint modeling frameworks. Early contributions by [40, 41] formalised the idea of linking longitudinal and survival processes via shared latent random effects, allowing both processes to be correlated through unobserved heterogeneity. Although originally conceived as a mechanism for improving survival prediction, these shared-parameter models are implicitly bidirectional in nature, since the random effects influence both the longitudinal trajectory and the hazard of the event simultaneously. The occurrence of the event, in turn, may truncate or structurally modify the longitudinal series, thereby generating feedback that cannot be accommodated by two-stage approaches in which the biomarker and survival models are fitted separately. [42] extended this framework by introducing flexible parametric survival submodels such as Weibull, Gompertz, and spline-based forms, which reduce the computational burden of repeated numerical integration and make joint modeling more tractable in practice.

More recent developments have sought to relax parametric assumptions and capture the non-linear dynamics that often arise in feedback systems. Liu et al. [43] proposed a Bayesian semiparametric joint model in which the longitudinal submodel for proportional data is handled via a logit-transformation and B-spline basis expansion, while the random effects distribution is modelled nonparametrically using a Dirichlet process mixture. This construction increases flexibility and accommodates heterogeneous population structures that cannot be represented by Gaussian random effects alone. Importantly, their work incorporated Bayesian Lasso penalisation for variable selection, highlighting the relevance of shrinkage methods when modeling high-dimensional biomedical data in which multiple feedback pathways may exist. In parallel, computationally efficient approximations to joint models have been proposed. The traditional two-stage modeling strategy, in which biomarker trajectories are estimated first and then included as covariates in the survival model, is known to introduce bias by failing to account for endogenous feedback. To mitigate this, [44] introduced the novel two-stage modeling (N2stgM) approach, which preserves much of the efficiency of the joint model but at substantially lower computational cost. Their simulation and empirical results demonstrated that N2stgM can approximate full joint modeling performance in many settings, offering a pragmatic solution for large-scale biomedical data analysis.

Beyond classical statistical formulations, deep-learning approaches such as Dynamic-DeepHit [20] and recurrent-network joint models [21] have demonstrated competitive predictive performance in very large cohorts by replacing parametric structures with flexible neural representations. These methods scale well computationally but typically sacrifice the explicit, mechanistically interpretable feedback structure and pointwise uncertainty quantification preserved by our framework. We view the two strands as complementary: when interpretability and rigorous inference about the magnitude and direction of feedback are required, the bidirectional joint model with semi-parametric links is preferable; when prediction in massive cohorts is the primary aim, neural alternatives may be more efficient.

Although joint modeling has been most widely applied in contexts involving time-to-event outcomes, the bidirectional problem is equally relevant for multiple longitudinal processes evolving together. A recent scoping review by [45] systematically catalogued joint models for non–time-to-event longitudinal data, demonstrating that such models improve efficiency, reduce bias, and handle missingness more effectively than separate analyses. This review emphasises that joint modeling is no longer restricted to survival contexts and should be considered whenever interdependent longitudinal processes may be present. Collectively, these developments illustrate that bidirectionality can be addressed either by embedding longitudinal and survival outcomes in a common latent structure, by explicitly modelling time-varying feedback functions through splines or Bayesian nonparametric approaches, or by employing computational approximations that balance feasibility with statistical rigour.

An important implication of these methodological advances is their relevance for individual-level dynamic prediction. Traditional survival models typically generate baseline risk predictions that do not incorporate evolving biomarker information. Joint models, and especially their bidirectional extensions, enable updating of an individual’s risk trajectory as new longitudinal data are collected, while also allowing for structural breaks caused by intermediate events such as relapse or hospitalization. This has direct utility in clinical decision-making, where treatment strategies may be adapted based on the current and projected trajectory of a patient. As noted by [29], dynamic prediction frameworks within joint modeling provide subject-specific survival probabilities conditional on an individual’s biomarker history, and incorporating bidirectional feedback further improves accuracy by reflecting the reciprocal influence of events on future biomarker values. For patients with multimorbidity, such as diabetes and cardiovascular disease or cancer with systemic complications, these models can be embedded in risk calculators or electronic health record systems to provide personalised and adaptive prognostic tools.

This work advances joint modeling in three complementary directions. First, by embedding semi-parametric association functions *f(· )* within a joint longitudinal–survival model, it accommodates non-linear, threshold, and saturation effects that are common in biomedicine (e.g., eGFR decline in chronic kidney disease, FEV$_1$ in COPD, PSA or tumour volume in oncology). Linear links, although convenient, can distort both effect estimates and predictions when the true relationship bends or plateaus. Second, our bidirectional extension captures mechanistic feedback whereby events reshape the post-event biomarker dynamics through level and slope shifts; the empirical gains in calibration and AUC observed in simulations demonstrate that explicitly modeling feedback prevents attenuation of risk signals that arises when post-event dynamics are ignored. Third, the framework is operationalised in a dashboard that couples model fitting with explainable outputs (spline or neural additive model partial effects), dynamic risk summaries, and exportable figures suitable for case review and quality improvement.

These results align with and extend the foundational literature on joint models and dynamic prediction for longitudinal and time-to-event data [5, 8–10, 29]. Whereas classical approaches emphasise linear associations and unidirectional influence of biomarkers on event hazards, recent reflections and methodological contributions have highlighted the clinical plausibility and statistical necessity of feedback mechanisms, particularly in multimorbid and intervention-rich care settings [8, 46]. Our findings add rigorous simulation evidence that the combination of flexible links and feedback yields tangible improvements in discrimination and calibration, with manageable computational overhead via Laplace or adaptive Gauss–Hermite quadrature approximations.

Several practical considerations warrant emphasis. Identifiability of flexible *f(· )* requires sufficient spread of $m_i(t)$ over time and careful regularisation; our GCV-tuned penalties and cross-validated neural additive models mitigate overfitting while preserving clinically relevant curvature. When the observation process is strongly informative, additional joint modeling of visit intensity may be required to avoid bias; we observed modest attenuation when ignoring a mild intensity–state coupling, consistent with prior reports. For competing risks, cause-specific modeling and the cumulative incidence representation yield interpretable, endpoint-specific risk summaries; nonetheless, the choice between cause-specific and subdistribution hazards should reflect the clinical question. Finally, computational scaling is facilitated by modular estimation (separate fits per endpoint with shared longitudinal components) and by exploiting sparse structures in spline bases; for Bayesian estimation, Hamiltonian Monte Carlo provides robust uncertainty quantification at higher cost.

### Limitations

We summarise here the principal limitations of the work; each is also acknowledged in context in the relevant subsection above.

*Single applied example.* The methodological framework is demonstrated on PBC only. Although the Background motivates oncology, nephrology, and cardiometabolic applications, these are not validated empirically in this paper. The reference-dataset section of the dashboard (Reference datasets, use-cases, and data structure section) describes how analogous workflows would be configured for those settings, and the simulation parameters in Data-generating mechanisms section are calibrated to span them, but a full applied demonstration in a second disease area is left to future work.

*Internal validation only.* As discussed above, the PBC AUC of 0.78 is from 5-fold cross-validation, not external validation. The framework’s external generalisability across different healthcare systems and eras remains to be established.

*Identifiability constraints.* Reliable estimation of *κ* requires sufficient post-event longitudinal observations; small or sparsely-sampled cohorts may not support full bidirectional feedback estimation (Bidirectional extension of joint models section).

*Computational cost.* Bayesian estimation via HMC scales super-linearly with *n* and remains expensive for very large EHR cohorts; the penalised-likelihood pathway is the recommended default at *n > 1000*.

*Conditional non-informativeness of observation and censoring.* The default estimation pipeline assumes that the visit process and censoring are non-informative given the model. Severe violations require joint modeling of the observation/censoring process, which is supported as an optional extension but is not invoked by default.

*Simple random sampling assumption.* The current framework does not accommodate complex survey designs (stratification, clustering, unequal weighting). Extensions outlined in Future directions section address this as future work.

### Future directions

#### Extension to complex sampling designs

In practice, multimorbidity datasets often arise from surveys or registries with complex sampling designs involving stratification, clustering, and unequal selection probabilities. The current JMbdirect framework assumes simple random sampling, and extending it to accommodate complex designs is an important direction for future work. Two complementary approaches are feasible. First, design-weighted pseudo-likelihood methods could incorporate sampling weights into the EM algorithm by replacing the standard log-likelihood contributions with weighted versions $w_i \log L_i(\theta )$, where $w_i$ denotes the inverse probability of selection for subject *i*; this approach has been successfully applied in weighted generalized estimating equations and survey-weighted Cox models [47, 48]. Second, the Bayesian estimation pathway could be extended by incorporating a sampling model that conditions on the design variables, effectively embedding the selection mechanism in the hierarchical structure [49]. For clustered data (e.g., patients nested within hospitals), the random-effects structure in our longitudinal submodel can be augmented with cluster-level random effects to account for intra-cluster correlation, and the survival submodel can incorporate shared frailty terms. These extensions would broaden the applicability of bidirectional joint models to population-representative cohort studies and national health surveys, where ignoring the sampling design may lead to biased variance estimates and invalid inference.

## Conclusions

This work advances joint modeling of longitudinal and survival data in three ways. First, semi-parametric association structures allow flexible capture of threshold, saturation, and non-linear effects that linear links cannot accommodate. Second, bidirectional extensions formally encode feedback from events into biomarker dynamics, reducing bias and improving calibration in dynamic prediction. Third, these innovations are implemented in the JMbdirect package and an interactive dashboard, ensuring accessibility for applied researchers and clinicians.

Our simulations and applied examples demonstrate that accounting for both feedback and flexible associations yields tangible gains in predictive performance and clinical interpretability. Limitations remain, including increased computational demands for Bayesian estimation and the need for sufficient data spread to identify flexible links. Future work will extend the framework to recurrent events with terminal competing risks, fusion of multiple biomarkers, accommodation of complex sampling designs arising from stratified or clustered survey data, external validation across multiple healthcare systems, and integration with deep-learning–based dynamic survival predictors, and joint modeling of observation intensity.

By aligning statistical inference with the biological reality of multimorbidity and disease progression, JMbdirect provides a rigorous and practical foundation for individualized risk prediction in modern precision medicine.

## Supplementary Information

Supplementary Material 1. Tables S1–S3

## Data Availability

No datasets were generated or analysed during the current study.

## Abbreviations

AUC: Area under the receiver operating characteristic curve
CIF: Cumulative incidence function
CKD: Chronic kidney disease
CVD: Cardiovascular disease
DGP: Data-generating process
EHR: Electronic health record
EM: Expectation–maximization
GCV: Generalized cross-validation
HMC: Hamiltonian Monte Carlo
IBS: Integrated Brier score
MC SE: Monte Carlo standard error
NAM: Neural additive model
PBC: Primary biliary cirrhosis
REML: Restricted maximum likelihood
RMSE: Root mean squared error
