# Supplementary material for: JMbdirect: joint modeling with semi-parametric link functions for bidirectional feedback in longitudinal-survival data
Source: BMC Med Res Methodol. 2026 Aug 1;26:230. doi: 10.1186/s12874-026-02955-5 (PMC13717975; doi:10.1186/s12874-026-02955-5)
Supplement: Supplementary file 1 [file 12874_2026_2955_MOESM1_ESM.pdf]

Electronic Supplementary Material

Supplementary Tables S1–S3

JMbdirect: Joint Modeling with Semi-Parametric Link Functions for Bidirectional Feedback in Longitudinal–Survival Data

Atanu Bhattacharjee

Table S1: Sensitivity of estimation and prediction to feedback magnitude at $n = 200$ and $n = 1000$ for the spline-with-feedback and NAM-with-feedback specifications (competing risks, non-linear true association; 1000 replicates).

Feedback	n	Spline, FB				NAM, FB			
		Bias($\hat{\kappa}_{k0}$)	Bias($\hat{\kappa}_{k1}$)	AUC@5	IBS(0–5)	Bias($\hat{\kappa}_{k0}$)	Bias($\hat{\kappa}_{k1}$)	AUC@5	IBS(0–5)
None	200	0.02	0.01	0.79	0.152	0.02	0.01	0.78	0.154
Moderate	200	0.05	0.03	0.79	0.152	0.06	0.03	0.78	0.154
Strong	200	0.04	0.02	0.81	0.146	0.04	0.02	0.80	0.148
None	1000	0.00	0.00	0.87	0.129	0.00	0.00	0.86	0.131
Moderate	1000	0.02	0.01	0.88	0.128	0.02	0.01	0.87	0.130
Strong	1000	0.01	0.00	0.89	0.124	0.01	0.01	0.88	0.126

FB, feedback; NAM, neural additive model; AUC@5, time-dependent area under the curve at a 5-year horizon; IBS(0–5), integrated Brier score over 0–5 years; $\hat{\kappa}_{k0}$, estimated post-event level shift; $\hat{\kappa}_{k1}$, estimated post-event slope change.

Table S2: Sensitivity of estimation to the spline smoothing parameter λ across simulation scenarios. The GCV-selected value tracks the oracle value to within 5% in all settings.

True λ	Fitted λ (GCV)	Relative difference
0.1	0.095	−5.0%
0.5	0.512	+2.4%
1.0	0.982	−1.8%
2.0	2.041	+2.1%
5.0	4.897	−2.1%

GCV, generalized cross-validation. Relative difference is computed as (fitted − true)/true.

Table S3: Performance comparison of the spline and NAM specifications under a sharp threshold true association function ($n = 500$, competing risks, moderate feedback; 1000 replicates).

Estimator	Bias(β_1)	AUC@5	IBS(0–5)	Coverage (95%)
Spline, FB	0.04	0.84	0.137	0.93
NAM, FB	0.03	0.86	0.134	0.94

FB, feedback; NAM, neural additive model. Under a sharp threshold mechanism the NAM specification attains a small advantage in discrimination and calibration relative to the penalized spline.
